# The origin of snakes: revealing the ecology, behavior, and evolutionary history of early snakes using genomics, phenomics, and the fossil record

**DOI:** 10.1186/s12862-015-0358-5

**Published:** 2015-05-20

**Authors:** Allison Y Hsiang, Daniel J Field, Timothy H Webster, Adam DB Behlke, Matthew B Davis, Rachel A Racicot, Jacques A Gauthier

**Affiliations:** Department of Geology and Geophysics, Yale University, New Haven, Connecticut 06520 USA; Department of Vertebrate Zoology, National Museum of Natural History, Smithsonian Institution, Washington, DC 20560 USA; Department of Anthropology, Yale University, New Haven, Connecticut 06520 USA; Yale Peabody Museum of Natural History, Yale University, New Haven, Connecticut 06520 USA

**Keywords:** Serpentes, Phylogeny, Ancestral state reconstruction, Divergence time estimation, Combined analysis, Fossil tip-dating

## Abstract

**Background:**

The highly derived morphology and astounding diversity of snakes has long inspired debate regarding the ecological and evolutionary origin of both the snake total-group (Pan-Serpentes) and crown snakes (Serpentes). Although speculation abounds on the ecology, behavior, and provenance of the earliest snakes, a rigorous, clade-wide analysis of snake origins has yet to be attempted, in part due to a dearth of adequate paleontological data on early stem snakes. Here, we present the first comprehensive analytical reconstruction of the ancestor of crown snakes and the ancestor of the snake total-group, as inferred using multiple methods of ancestral state reconstruction. We use a combined-data approach that includes new information from the fossil record on extinct crown snakes, new data on the anatomy of the stem snakes *Najash rionegrina, Dinilysia patagonica*, and *Coniophis precedens,* and a deeper understanding of the distribution of phenotypic apomorphies among the major clades of fossil and Recent snakes. Additionally, we infer time-calibrated phylogenies using both new ‘tip-dating’ and traditional node-based approaches, providing new insights on temporal patterns in the early evolutionary history of snakes.

**Results:**

Comprehensive ancestral state reconstructions reveal that both the ancestor of crown snakes and the ancestor of total-group snakes were nocturnal, widely foraging, non-constricting stealth hunters. They likely consumed soft-bodied vertebrate and invertebrate prey that was subequal to head size, and occupied terrestrial settings in warm, well-watered, and well-vegetated environments. The snake total-group – approximated by the *Coniophis* node – is inferred to have originated on land during the middle Early Cretaceous (~128.5 Ma), with the crown-group following about 20 million years later, during the Albian stage. Our inferred divergence dates provide strong evidence for a major radiation of henophidian snake diversity in the wake of the Cretaceous-Paleogene (K-Pg) mass extinction, clarifying the pattern and timing of the extant snake radiation. Although the snake crown-group most likely arose on the supercontinent of Gondwana, our results suggest the possibility that the snake total-group originated on Laurasia.

**Conclusions:**

Our study provides new insights into when, where, and how snakes originated, and presents the most complete picture of the early evolution of snakes to date. More broadly, we demonstrate the striking influence of including fossils and phenotypic data in combined analyses aimed at both phylogenetic topology inference and ancestral state reconstruction.

**Electronic supplementary material:**

The online version of this article (doi:10.1186/s12862-015-0358-5) contains supplementary material, which is available to authorized users.

## Background

Living snakes (Serpentes) comprise more than 3,400 species. They are virtually cosmopolitan in distribution, occupying fossorial, arboreal, terrestrial, and aquatic environs, and living in climates ranging from arid deserts to the open ocean. Crown snakes are split into two major clades: Scolecophidia, which includes blind snakes and thread snakes, and Alethinophidia, which comprises all other snakes [1]. Within Alethinophidia, the most diverse and disparate clade is Henophidia, which includes booids (pythons and boas) and caenophidians (viperids, elapids, and colubrids).

The ecological and evolutionary origins of snakes have long been debated in light of the clade’s incredible extant diversity, and the distinctive snake body plan. Among the major questions surrounding snake origins are whether snakes first arose on the Mesozoic supercontinent of Gondwana or Laurasia, whether snakes originated on land or in the sea, and whether the earliest snakes were fossorial, terrestrial, or arboreal in their habits. Inferring the phenotype, ecology, and biogeography of the ancestral snake has heretofore been hindered by the relative lack of informative fossils of early stem snakes. Furthermore, deciphering the evolutionary origins of snakes is complicated by the fact that scolecophidian snakes, which are sister to all other crown snakes, are highly modified and overprinted with unique morphological and behavioral apomorphies [2,3]. These include ecological and behavioral features such as exclusively fossorial habits, specialized feeding on social insects and their larvae, as well as derived phenotypic characteristics such as highly reduced eyes, uniquely modified jaws, and smooth, deeply imbricate, cycloid body scales.

However, recent discoveries of more complete, better-preserved specimens of fossil stem snakes such as *Dinilysia patagonica* (Santonian-Campanian) [4], *Najash rionegrina* (Cenomanian) [5,6], and *Coniophis precedens* (Maastrichtian) [7] suggest that the unique characteristics of scolecophidians likely do not represent the ancestral condition for snakes. Phylogenetic analyses indicate that *Dinilysia*, *Najash*, and *Coniophis* represent successively more remote hierarchical sisters to crown snakes, with *Dinilysia* representing the immediate sister to the crown [4,7,8]. These specimens thus provide abundant new data on the origin of early snakes. Importantly, these fossil species are also unambiguously terrestrial [4,7,8]: this, in combination with the recently revised phylogenetic position of the limbed Tethyan marine snakes (Simoliophiidae; e.g., *Haasiophis terrasanctus* [9]*, Eupodophis descouensis* [10]*,* and *Pachyrachis problematicus* [11]) as nested within Alethinophidia (rather than representing stem snakes) [4,8], offers compelling evidence against the marine origin hypothesis for snakes.

These recent fossil findings, in conjunction with fossils of previously unknown, extinct members of crown Serpentes such as *Sanajeh indicus* [12] and *Kataria anisodonta* [13], provide abundant new data on the morphology and evolution of the earliest known snakes, and emphasize the crucial role fossils play in accurately inferring evolutionary history [14]. In light of this newfound wealth of fossil data, we infer the ecology, behavior, and biogeography of early snakes by synthesizing information from the fossil record with phenotypic and genetic data for Recent species. Specifically, we reconstruct the ancestor of the snake total-group and of crown snakes, using both established and recently developed analytical methodologies. Additionally, we infer divergence time trees using a combination of traditional node-based dating and novel fossil tip-dating methods [15,16] to explore the pattern and timing of major events in early snake evolution.

## Results and discussion

### Phylogenetic analyses

**Figure 1:**
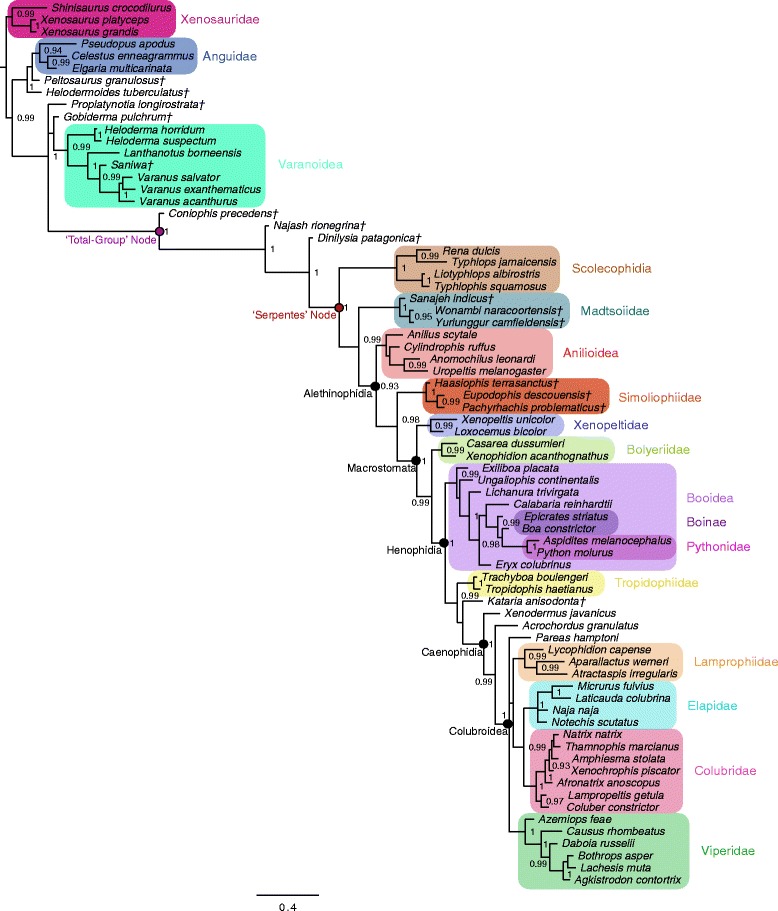
Bayesian phylogenetic tree inferred from phenotypic dataset. Fifty-percent majority rule consensus tree from Bayesian analysis of the state-partitioned phenotypic dataset (766 characters) under the Mkv model [74] in *MrBayes*. Node values are Bayesian posterior probability support values; only values above 90% are shown. Scale bar represents substitutions/site. Colored boxes indicate major clades. Fossil taxa are marked with a dagger (†).

**Figure 2:**
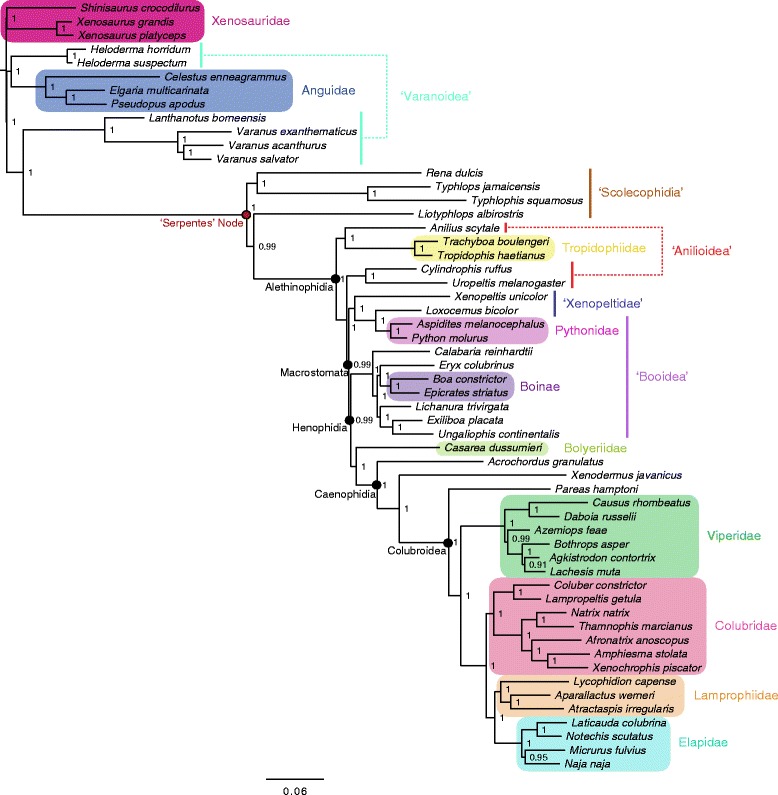
Bayesian phylogenetic tree inferred from genetic dataset. Fifty-percent majority rule consensus tree from Bayesian analysis of the gene-partitioned genetic dataset (21 nuclear loci and one mitochondrial locus) in *MrBayes*. Node values are Bayesian posterior probability support values; only values above 90% are shown. Scale bar represents substitutions/site. Colored boxes indicate major clades. Colored lines indicate major clades from traditional taxonomies that do not resolve as monophyletic groups in this topology.

**Figure 3:**
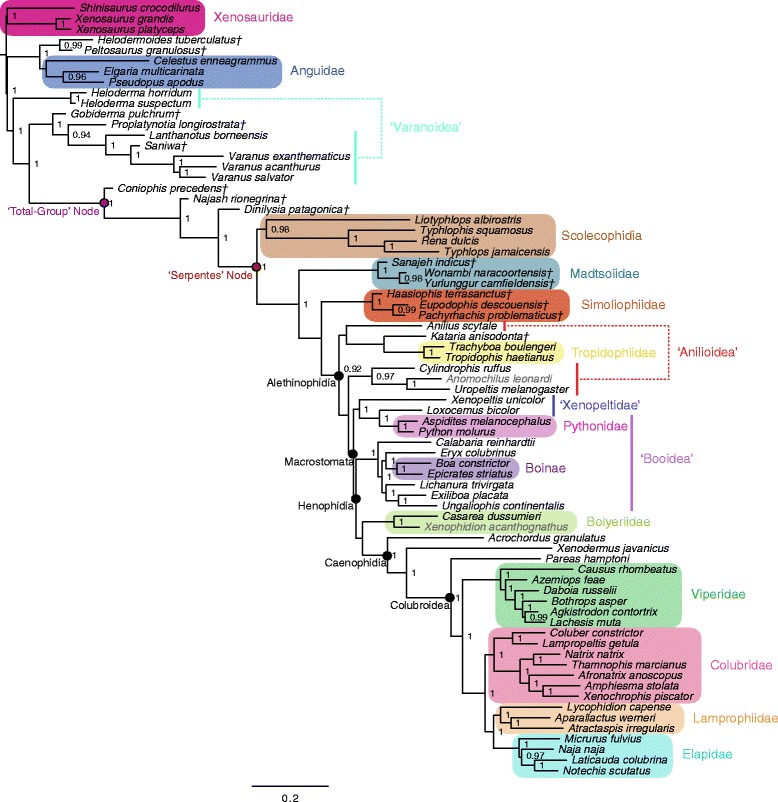
Bayesian phylogenetic tree inferred from combined genetic and phenotypic dataset, unconstrained. Fifty-percent majority rule consensus tree from Bayesian analysis of the unconstrained combined genetic and phenotypic datasets (with corresponding partition schemes) in *MrBayes*. Node values are Bayesian posterior probability support values; only values above 90% are shown. Scale bar represents substitutions/site. Colored boxes indicate major clades. Colored lines indicate major clades from traditional taxonomies that do not resolve as monophyletic groups in this topology. Fossil taxa are marked with a dagger (†). Grayed taxon names indicate extant species that are included on the basis of phenotypic data only.

**Figure 4:**
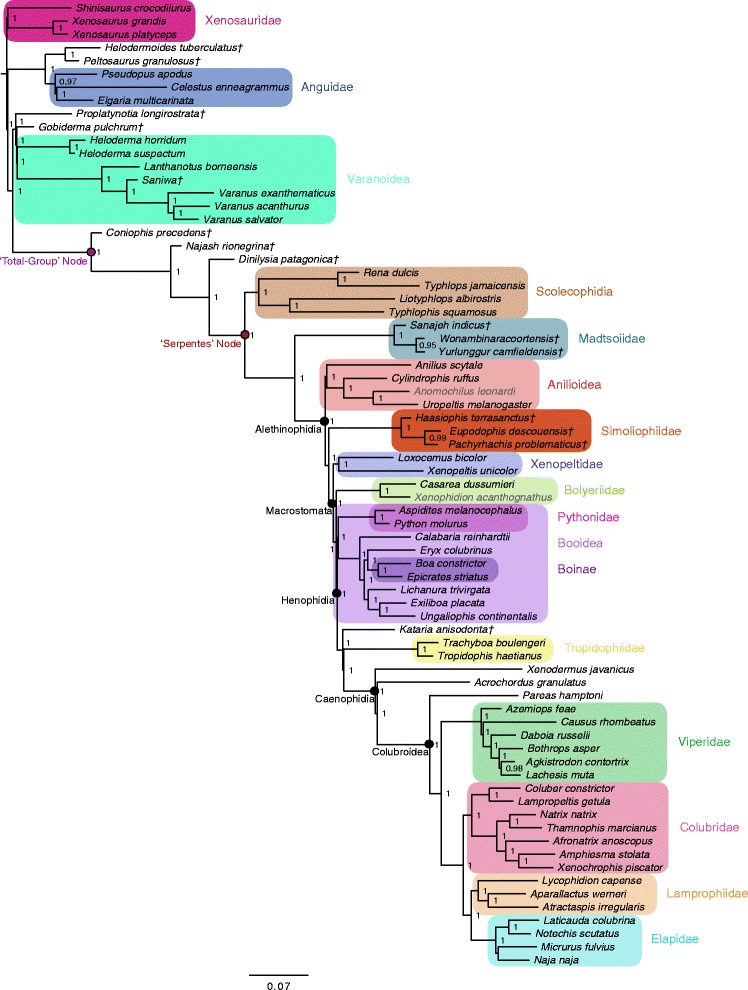
Bayesian phylogenetic tree inferred from combined genetic and phenotypic dataset, constrained. Fifty-percent majority rule consensus tree from Bayesian analysis of the combined genetic and phenotypic datasets, with topology constraints implemented as described in the text, as estimated in *MrBayes*. Node values are Bayesian posterior probability support values; only values above 90% are shown. Scale bar represents substitutions/site. Colored boxes indicate major clades. Fossil taxa are marked with a dagger (†). Grayed taxon names indicate extant species that are included on the basis of phenotypic data only.

**Figure 5:**
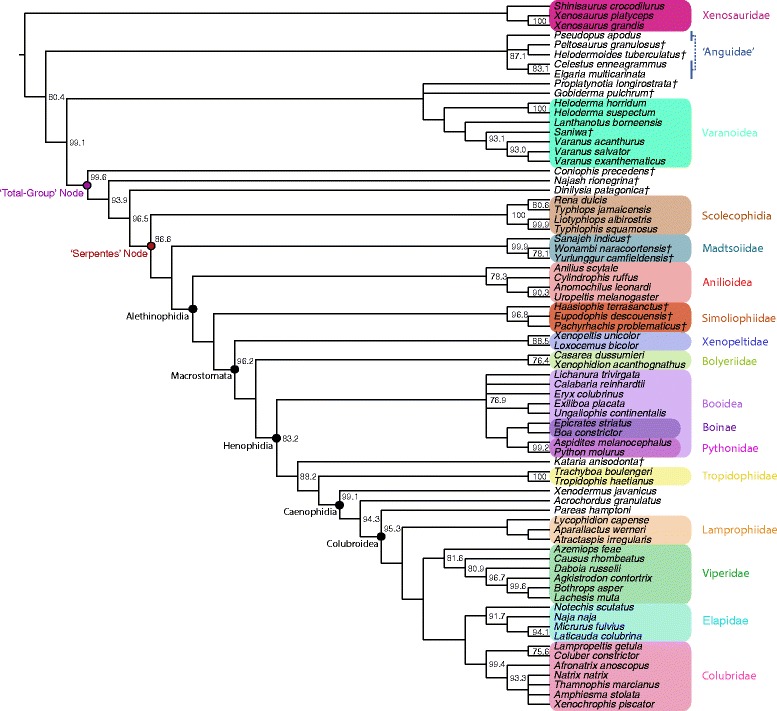
Phylogenetic tree inferred using parsimony using phenotypic data. Fifty-percent majority rule bootstrap consensus tree from heuristic searches under the parsimony framework using the complete phenotypic dataset. Node values are bootstrap probabilities; only those above 75% are shown. Colored boxes indicate major clades. Colored lines indicate major clades from traditional taxonomies that do not resolve as monophyletic groups in this topology. Fossil taxa are marked with a dagger (†).

**Figure 6:**
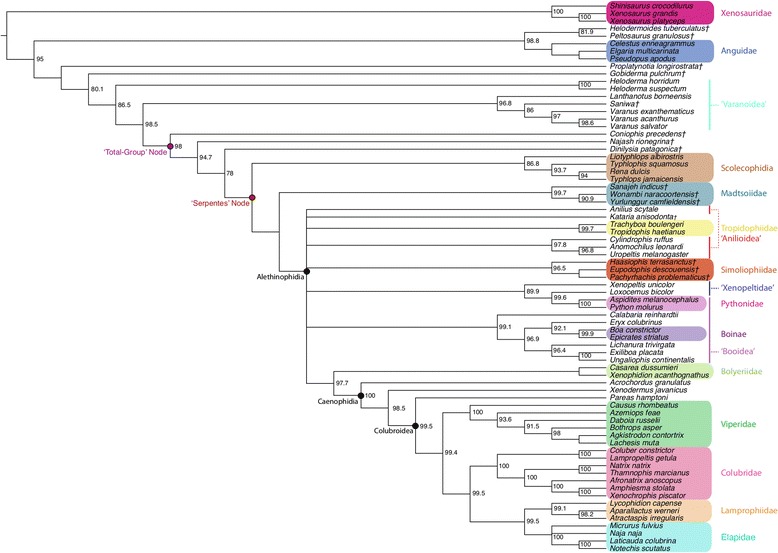
Phylogenetic tree inferred using parsimony using the combined dataset. Fifty-percent majority rule bootstrap consensus tree from heuristic searches under the parsimony framework using the combined (phenotypic + genetic) dataset. Node values are bootstrap probabilities; only those above 75% are shown. Colored boxes indicate major clades. Colored lines indicate major clades from traditional taxonomies that do not resolve as monophyletic groups in this topology. Fossil taxa are marked with a dagger (†).

The complete dataset comprises 766 phenotypic characters, 18,320 base pairs (bp) from 21 nuclear loci and one mitochondrial locus, and 11 novel characters for ancestral state reconstruction (see Methods for more details). Bayesian phylogenetic trees were inferred using the following four datasets: 1) phenotypic data alone (hereafter referred to as the ‘phenotypic’ topology; Figure 1); 2) genetic data alone (hereafter referred to as the ‘genetic’ topology; Figure 2); 3) the combined phenotypic and genetic dataset, without any topological constraints (hereafter referred to as the ‘unconstrained’ topology; Figure 3); and 4) the combined phenotypic and genetic dataset, with topological constraints enforced such that the relationships of the major clades correspond to those inferred using the morphological data (hereafter referred to as the ‘constrained’ topology; Figure 4). The constrained analysis was implemented in order to test hypotheses of character evolution on the phenotypic tree topology with branch lengths inferred using the complete dataset. In addition, maximum parsimony trees were inferred using the phenotype-only dataset (Figure 5) and the combined dataset (Figure 6).

In general, most nodes are consistent across all trees with high support. Clades that appear in both the tree inferred from the phenotypic dataset using parsimony and the tree inferred using Bayesian methods are always well supported under both optimality criteria. In the few instances where parsimony and Bayesian topologies for the phenotypic dataset differ, support for an alternative topology is invariably poor (e.g., support for monophyly of Boinae [*Epicrates striatus + Boa constrictor*] is strong in parsimony [Figure 5], while support for paraphyly of Boinae is weak in the Bayesian analysis [Figure 1]). The parsimony and Bayesian analyses of the (unconstrained) combined dataset also match in general, with the one notable exception of parsimony inferring a large polytomy at the base of Alethinophidia, comprised of *Anilius*, *Kataria*, Tropidophiidae, the clade of *Cylindrophis* + *Anomochilus* + *Uropeltis*, the Simoliophiidae, the clade of *Xenopeltis* + *Loxocemus* + Pythonidae, and a polyphyletic ‘Booidea’ (Figure 6).

The most striking differences are between trees inferred from the phenotype vs. the genotype. Scolecophidia, for example, is inferred to be paraphyletic (and Anomalepididae polyphyletic) in the genetic tree (Figure 2). This result concurs with other recent phylogenetic analyses using genetic data to target snake interrelationships [17-19]. However, in the phenotypic tree (Figure 1), as well as in the combined trees (both unconstrained [Figure 3] and constrained [Figure 4]), Scolecophidia is inferred to be the monophyletic sister to Alethinophidia (however, in the phenotypic and constrained topologies, a sister relationship between the scolecophidian clades Leptotyphlopidae and Typhlopidae is strongly supported, whereas in the unconstrained topology, the anomalepidids *Typhlophis squamosus* and *Liotyphlops albirostris* are successive sisters to Typhlopidae, rendering Anomalepididae paraphyletic). It is particularly notable that the unconstrained tree in our study recovers a monophyletic Scolecophidia, as it suggests that the addition of phenotypic data to a dataset dominated by genetic data (as would typically be the case in phylogenetic analyses that combine the two sources of data) can have significant effects on tree topology – in this case, resulting in the more traditional inference of scolecophidian monophyly.

Several other major differences exist between the phenotypic/constrained trees and the genetic/unconstrained trees. The viperid snake *Daboia russelii* is inferred to be sister to Crotalinae (i.e., pit vipers; in this case, *Bothrops* + *Agkistrodon* + *Lachesis*) in the phenotypic (Figure 1), unconstrained (Figure 3), and constrained trees (Figure 4), whereas it is sister to *Causus rhombeatus* in the genetic tree (Figure 2), making it part of a clade that is sister to all other Viperidae. In addition, the phenotypic and constrained trees strongly support a monophyletic Xenopeltidae (= *Xenopeltis unicolor + Loxocemus bicolor*) as sister to all other members of Macrostomata, whereas the genetic and unconstrained trees firmly place them as successive sisters to Pythonidae (which is also recovered to be outside Booidea). This result concurs with other recent studies of snake phylogeny based on concatenated gene sequences [18].

The position of Tropidophiidae within Alethinophidia is radically different among trees derived from these datasets. In the genetic and unconstrained trees, Anilioidea is polyphyletic (Figures 2 and 3): *Cylindrophis*, *Uropeltis*, and *Anomochilus* form a clade sister to Macrostomata, with *Anilius* + Tropidophiidae (= *Trachyboa boulengeri* + *Tropidophis haetianus*) as the next successive sister group. The *Anilius* + Tropidophiidae clade, also termed Amerophidia [20] after their exclusive extant presence in the New World (though, notably, the earliest total-group tropidophiids are known from Europe and North Africa in the late Eocene [21-25]), is strongly supported by molecular data in this study, in agreement with previous phylogenomic analyses of snake phylogeny [1,19,26,27]. In contrast, the phenotypic and constrained trees recover Tropidophiidae in its traditional position nested within henophidian macrostomatans, as sister to Caenophidia (Figures 1 and 4). Notably, in the unconstrained tree, the support values for the *Anilius* + Tropidophiidae clade, and for the sister relationship between the clade of *Cylindrophis + Uropeltis + Anomochilus* and Macrostomata, are not significant. This collapse in support values relative to the genetic tree (for which the posterior probabilities for both hypotheses are 100%) is likely due to the inclusion of strongly discordant phenotypic data in the unconstrained analysis. Indeed, to date only a single morphological apomorphy – specifically, an oviduct connecting with diverticuli of the cloaca, instead of directly with the cloaca as in all other squamates – has been found to be shared by *Anilius* and Tropidophiidae [28]. In addition, the splitting of *Cylindrophis*, *Uropeltis*, and *Anomochilus* from *Anilius* in the genetic tree, and the placement of the former three taxa within basal macrostomatans, would require the “redevelopment of a complex multipinnate jaw adductor musculature comparable to that of lizards” [29]. Furthermore, although it can be argued that *Anilius* and uropeltines may be artificially drawn together due to convergence in their skulls related to shared fossorial habits, such an argument does not account for the fact that other fossorial snakes, such as *Loxocemus*, are never recovered as being closely allied to *Anilius* or *Uropeltis*. The question of whether Amerophidia (*Anilius* + Tropidophiidae) represents a true clade clearly requires further study; regardless, including phenotypic characters in our combined dataset collapses strong support for Amerophidia, again demonstrating the potential influence of including phenotypic data even in large-scale phylogenomic studies, despite marked discrepancies in the number of characters from each source (in this case, 18,320 nucleotide bp vs. 766 phenotypic characters). We emphasize here that we do not mean to suggest that the morphological signal is necessarily the ‘correct’ one, but rather that including morphological data can be beneficial and effective at identifying portions of phylogenetic trees that may not be as uncontroversial as genomic data alone may suggest – whether by directly affecting the topology itself (as in Scolecophidia becoming monophyletic in our unconstrained combined analysis) or by collapsing the support values of controversial groups (as in the case of Amerophidia). Our study presents empirical evidence against the commonly held view that genomic data, by virtue of their abundance, will inevitably ‘swamp out’ conflicting signals from morphological data, rendering their contribution negligible and thus ignorable (viz., that phenotypic characters are merely baubles to be suspended on genomic trees).

The placement of several fossil taxa differs between the unconstrained tree and the phenotypic and constrained trees. For instance, marine simoliophiids are inferred to form a clade that is sister to Alethinophidia in the unconstrained tree (Figure 3), in contrast to the phenotypic (Figure 1) and constrained trees (Figure 4), where they are nested within Alethinophidia as sister to crown Macrostomata. The Simoliophiidae + Alethinophidia sister relationship in the unconstrained tree is, however, poorly supported. In all cases, simoliophiids are inferred to be nested within crown snakes with high support, and do not represent stem snakes (as has been suggested by some [11]), despite retaining tiny hindlimbs.

The unconstrained, constrained, and phenotypic trees all strongly support Madtsoiidae (= *Sanajeh indicus*, *Wonambi naracoortensis*, and *Yurlunggur camfieldensis*) as stem alethinophidians (Figures 1, 3, and 4), and thus as belonging to the snake crown-group (see also Apesteguía and Zaher [30] and Longrich *et al.* [7]). This suggests that madtsoiids, and by extension the ancestor of crown snakes, likely also retained tiny hindlimbs with ankles and toes, as in stem snakes and simoliophiids – unlike any extant snakes. At this point, however, we can only be sure that madtsoiids retained at least part of the hindlimb, as *Wonambi naracoortensis* has a scolecophidian-like triradiate pelvis with a well-developed acetabulum for reception of the femoral head [31].

The unconstrained tree and constrained/phenotypic trees further differ in the placement of the Paleocene fossil snake *Kataria anisodonta*, from South America [13]. In the unconstrained topology, *Kataria* is reconstructed as sister to Tropidophiidae, with *Anilius scytale* as sister to both of these taxa (Figure 3). In contrast, the phenotypic and constrained analyses (Figures 1 and 4) infer that *Kataria* is nested within Macrostomata and Henophidia as sister to Tropidophiidae + Caenophidia, in agreement with Scanferla *et al.* [13]. The placement of *Kataria* exhibited in the unconstrained tree is not strongly supported, and likely reflects a passive consequence of its allegiance with Tropidophiidae and Caenophidia, clades that are strongly supported in all analyses of all datasets.

Both the genetic and unconstrained trees resolve *Xenodermus javanicus* as the immediate sister taxon of Colubroidea, followed by *Acrochordus granulatus* as sister to the *Xenodermus* + Colubroidea clade (Figures 2 and 3). This is contrary to the strongly supported phenotypic/constrained topology, in which the positions of *Xenodermus* and *Acrochordus* are reversed (Figures 1 and 4). Although other studies of concatenated gene sequences have inferred the same topology as our genetic tree with equally high support [18,26], the recent Pyron *et al*. [19] supertree recovered an alternate, highly supported topology in which Xenodermatidae and *Acrochordus* form a clade that is sister to Colubroidea. The disagreement between genetic tree topologies for these taxa illustrates the existence of extensive homoplasy in multi-gene, phylogenomic datasets [32,33] (‘homoplasy’ here intended in the broad sense as referring to any potentially confounding phylogenetic signal that does not arise from common ancestry – circumscribing not merely functional convergence but also phenomena such as long-branch attraction and incomplete lineage sorting). Definitively unraveling the relationships among Xenodermatidae, *Acrochordus*, and Colubroidea will require further study.

In all analyses, *Dinilysia patagonica, Najash rionegrina,* and *Coniophis precedens* form successive sisters to crown Serpentes, supporting their status as early members of Pan-Serpentes, with *Coniophis* as the earliest-diverging stem snake currently known. Although *Najash* and *Coniophis* are clearly stem snakes more distantly related to the crown than is *Dinilysia*, the inference that *Coniophis*, rather than *Najash*, is sister to all other known snakes depends on the correct attribution to that species of isolated, tooth-bearing bones with numerous disarticulated vertebrae, all from the Maastrichtian of the American Interior West [7]. The validity of this standpoint remains controversial, and its resolution will require additional discoveries of associated/articulated *Coniophis* specimens [34] and more complete knowledge of *Najash*.

### Ancestral state reconstruction

Ancestral state reconstructions (ASR) were conducted for 11 characters (see Additional file 1) for the genetic (see Additional file 2), unconstrained (see Additional file 3), and constrained (see Additional file 4) topologies, using three methods: parsimony, Yang *et al.*’s maximum likelihood (ML) re-rooting method [35], and Bayesian stochastic character mapping [36,37], representing a total of 99 individual ASR analyses. We chose to implement all three methods in order to compare their results and establish robustness (or the lack thereof) of our results. In particular, we were concerned with how variable reconstructions were across methods, and how different ways of defining a character – i.e., the binary ‘Plate I’ character vs. the much more highly atomized ‘Plate II’ character – might affect our results. The Bayesian stochastic character mapping method was chosen in particular for its ability to include polymorphic and missing characters – which are extensive in our dataset – during the inference process. ASR results are reported for the ‘Serpentes’ (i.e., crown-snake) node and the ‘Total-Group’ node, with Bayesian results reported as posterior probabilities (PP), ML results reported as proportions of total likelihood (PTL), and the most parsimonious state(s) reported for parsimony.

ASR results are largely invariant across different reconstruction methods and tree topologies. However, several reconstructions fail (i.e., produce ambiguous/uninformative results where all possible states are equally likely) for the genetic topology. Specifically, this occurs for the ‘Diel’, ‘Plate II’, ‘Biome’, ‘Habitat Stratum’, and ‘Aquatic Habits’ characters. In contrast, for the constrained and unconstrained tree topologies, ASR fails only for the ‘Biome’ character using the ML method. This suggests that this genetic topology is particularly poorly suited to ancestral state analyses, perhaps because, due to its lack of intermediate, branch-shortening fossils, it fails to approximate the full distribution of character states that existed across the evolutionary history of snakes. This underscores the importance of including fossils as terminal taxa in ancestral state reconstruction analyses; for scenarios in which the genetic-only dataset fails in its ancestral state reconstruction, analyses of the combined datasets fail in only one of these (the highly variable ‘Biome’ character, using ML). Previous studies, both theoretical [38-40] and empirical [14,41-43], have demonstrated that the inclusion of fossil data in ancestral state reconstructions improves the precision, accuracy, and overall performance of these analyses. Our results corroborate these ideas, further demonstrating that in certain cases, the lack of fossil taxa in these analyses may actually render the reconstruction of ancestral states impossible. Fossil data are indispensable for reliably interpreting evolutionary history, as they serve to constrain possible hypotheses of character evolution and capture a more complete picture of character state distributions across evolutionary time and phylogenetic diversity.

Both the ancestor of crown snakes and the earliest known ancestor of the snake total-group are reconstructed unambiguously by all methods and on all topologies to have been land-dwelling, supporting the hypothesis that snakes originated in a terrestrial, rather than a marine, setting [4,7,8]. This is consistent with independent inferences of terrestrial habits for the oldest member of Pan-Serpentes (late Upper Albian) [44], *Najash rionegrina*, *Dinilysia patagonica* [45], and *Lapparentophis defrennei* [46]. These results further corroborate the suggestion that the limbed Tethyan Simoliophiidae represent an independent invasion of the marine realm. Although a terrestrial origin of snakes might imply that the snake body plan (e.g., reduced limbs and long bodies) is an adaptation for a burrowing lifestyle (fossoriality) [47], our inference for the primary habitat stratum for both the ‘Serpentes’ and the ‘Total-Group’ node is somewhat ambiguous: although the Bayesian and ML methods reconstruct the most likely stratum for both ancestors as surface-dwelling, the PP and PTL values are relatively low in the constrained topology (around 0.70 to 0.80, rather than > 0.90 as in most of the other reconstructions). Furthermore, reconstructions of terrestrial habits and fossoriality are equally parsimonious for both ancestors and topologies. Such ambiguity is not entirely unexpected, as many extant snakes exhibit a combination of habits, and some species may even vary in stratum preference based on age and size [48]. Regardless, our results suggest that the conclusion that the snake body plan evolved as an adaptation for a fossorial lifestyle is by no means foregone, and that burrowing taxa such as scolecophidians, and perhaps even anilioids, may have evolved from ancestors less committed to life underground [49].

Several additional conclusions can be drawn regarding the ecology and behavior of both the ancestor of crown snakes, and the ancestor of total-group snakes based on our analyses. Both ancestors likely inhabited well-vegetated environs in warm, moist, and equable climates (characterized as tropical to subtropical broad-leafed forest biomes today; note, however, that broad-leafed evergreen forests did not exist in the middle Cretaceous, and that these ‘biome’ characters refer to analogous physical climate conditions, regardless of the specific plants that happen to live in them today). This ecological preference spans much of the early history of snakes, from the branch stretching from Serpentes to Caenophidia. This may explain why, despite an extraordinary diversity of squamate fossils from these sediments, snakes have never been recovered from the more arid environs of the Upper Cretaceous of Mongolia [50].

**Figure 7:**
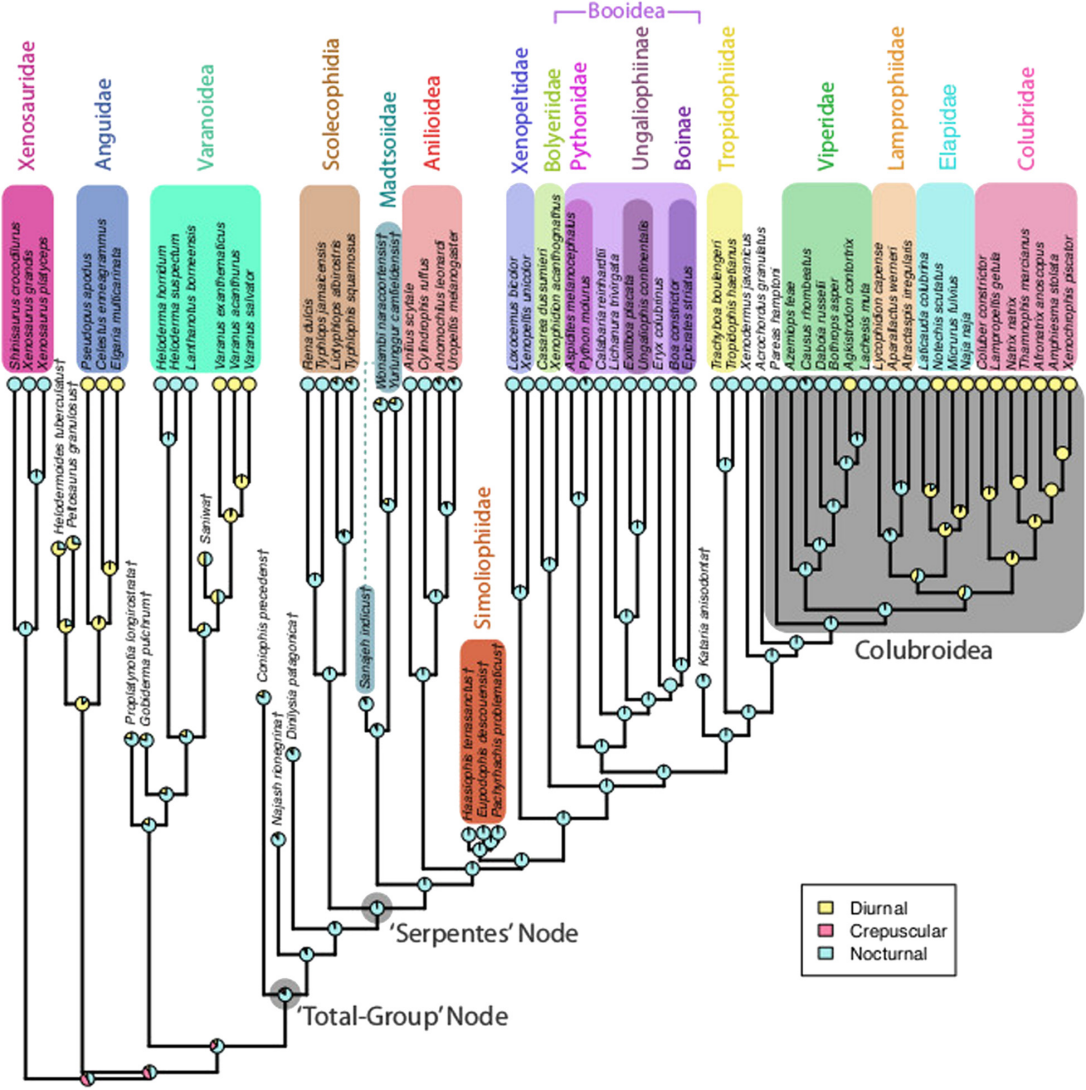
Ancestral state reconstruction of diel activity pattern. Bayesian SIMMAP ancestral state reconstruction using the constrained tree for the history of the ‘Diel Activity Pattern’ character. Nocturnality is inferred to be ancestral for snakes. The grey box marks the clade Colubroidea, within which diurnal habits re-evolved. Note that SIMMAP estimates the most likely states for tip taxa that are coded as missing or polymorphic – as such, some of the tip states exhibited in this figure are *inferred* tip states, not *coded* tip states (e.g., the fossil taxa were coded as missing data; the states they exhibit here are states inferred by the SIMMAP method). Fossil taxa are marked with a dagger (†).

Ancestral snakes are strongly inferred to have been nocturnal, with the acquisition of diurnal habits apparently occurring inside Colubroidea, specifically in the clade stemming from the last common ancestor of Elapidae and Colubridae (Figure 7). This return to diurnal habits – which are likely ancestral for reptiles [51] – may explain certain aspects of the evolutionary history of Colubroidea. Specifically, although colubroids experienced extensive diversification during Late Oligocene climatic warming, this wide taxonomic breadth was not matched with high relative abundance (compared to other snakes such as booids) until the latter half of the Miocene, when colubroids became dominant in the cooler and drier habitats that emerged at higher latitudes [52,53]. The success of the Colubroidea in these higher-latitude environments may have been facilitated by the reemergence of diurnality within that lineage, as colder nighttime temperatures may have limited nocturnal activity for ectothermic snakes.

The feeding behavior of the earliest snakes was inferred to have been similar to that of most extant snakes: they were likely widely foraging stealth predators, hunting soft-bodied prey subequal to head size (likely small vertebrates, either while active, e.g., nocturnal mammals, or asleep, e.g., diurnal squamates). Constriction is relatively restricted in its phylogenetic distribution, and likely did not arise outside of crown Alethinophidia.

The Bayesian and likelihood reconstructions reported above are all supported by parsimony ASR for all three topologies of interest, with the exception of prey preference, where parsimony reconstructs both soft-bodied prey and termites/ants (including their larvae and eggs) as being equally parsimonious on the genetic topology. This is likely due to a combination of the lack of fossil taxa in the genetic topology and the position of the termite-/ant-eating scolecophidians as sister to all other extant snakes. Virtually all reconstructions are highly supported (i.e., > 0.90 PP and PTL) by Bayesian and ML methods across the combined tree topologies, with the exception of the ‘Biome’ character for both topologies (for which the ML ASR fails), the ‘Constriction’ character for both topologies (for which the absence of constriction at the ‘Serpentes’ and ‘Total-Group’ nodes still exhibits the highest PP and PTL, but with values less than 0.90, but greater than 0.80), and the ‘Habitat Stratum’ character for the constrained topology (for which the surface-dwelling ‘terrestrial’ reconstruction exhibits PP and PTL values less than 0.90, but greater than 0.70). All successful ASR analyses for the genetic topology exhibit PP and PTL values greater than 0.80, with the exception of the ‘Diel’ character, for which the PP value of nocturnal habits, the most highly supported reconstruction, is 0.6610 (see Additional file 2).

Lagrange [54] biogeographic analysis of the ‘Plate II’ tectonic plate character yielded ambiguous results (see Additional file 5) for all tree topologies when the complete dataset (hereafter, the ‘full-genus’ distribution – whereby tip taxa were coded to represent the entire biogeographic range of the genus to which they belong in traditional taxonomies – see Methods) was used. When the taxa in the analysis were instead coded to reflect only the biogeographic ranges of individual species (‘no-genus’ distribution), Lagrange infers that both the ancestor of crown snakes and the ancestor of the snake total-group likely originated on Laurasia for the constrained tree (74.70% North America, 13.12% Asia, and 12.18% for North America + South America for the total-group node; 63.65% North America, 18.13% North American + Asia, 10.92% South America + Asia, 9.94% Asia, and 7.37% South America for the crown-snake node). For the unconstrained tree, the results are equivocally split between Laurasian and Gondwanan origins (32.72% North America, 32.65% North America + South America, 16.98 Asia, 9.92% South America + Asia, and 7.72% for North America + Asia for the total-group node; 37.64% South America, 34.16% South America + Asia, 13.83% North America, 9.25% for North America + Asia, and 5.11% for North America + South America for the crown-snake node). In contrast, the ancestor of crown snakes is unequivocally reconstructed as having originated on Laurasia for the genetic tree using the no-genus distribution (86.45% North America and 13.55% North America + Asia). However, the Laurasian reconstruction for the ancestor of crown snakes using the no-genus distribution is potentially influenced by sampling bias, as our dataset contains mostly representatives of Scolecophidia from North America and the Caribbean, despite the worldwide distribution of scolecophidians. This phenomenon, resulting in more complete genetic data for Nearctic scolecophidians than for other biogeographic zones, is likely due to the relative ease of access to these sampling localities for researchers hailing from the Northern Hemisphere.

The inability of the Lagrange method to produce an unambiguous result suggests that such biogeographic methods, which require introducing sources of uncertainty (e.g., constructing a relative dispersal probability matrix, for which there is no clear standard), may not be ideal for reconstructing dispersal history across long stretches of geological time. This is likely due in no small part to the breakdown of the conceptual foundations of these methods when geographical areas – which must necessarily be predefined to create dispersal probability matrices – change significantly through time (e.g., although the modern-day continent of Africa belonged to the Mesozoic supercontinent of Gondwana, it is unclear how reliably we can reconstruct dispersal history to and from ‘Africa’ when its modern-day identity was largely irrelevant during the Mesozoic – particularly when ambiguity abounds both in terms of subjective ‘dispersal probabilities’ and the estimated position through time of the landmass we call ‘Africa’ due to continental drift). This issue is likely aggravated when the organisms under consideration are highly dispersive, and thus likely to make biogeographic leaps that might seem extremely unlikely *a priori*.

Several lines of evidence suggest that snakes, particularly relative to other squamate reptiles, are particularly adept dispersers: 1) snakes have been empirically demonstrated to exhibit larger ranges than non-snake lizards [55,56] – when only terrestrial species are taken into consideration, snakes exhibit median range sizes that are ~4.5 times larger than that of non-snake lizards [57]; 2) hydrophiine snakes are unique among extant squamates in being adapted exclusively for marine lifestyles (in contrast to the iguanian *Amblyrhynchus cristatus*, which forages in the ocean while living primarily on land), demonstrating the remarkable capacity of Serpentes to adapt to and inhabit environments that traditionally hinder the dispersal of terrestrial organisms. This idea is corroborated by snakes having invaded aquatic (e.g., natricines, homolopsines, calamariines, *Acrochordus*) and marine habitats (e.g., hydrophiines, simoliophiids, palaeophiids) multiple times in their evolutionary history, perhaps facilitated by natural floatation conferred by enlarged right lungs that extend down the body, as well as a style of terrestrial locomotion that approximates anguilliform-style swimming; and 3) the biogeographic ranges of certain snake clades suggest dispersal capabilities across large stretches of water. For instance, *Candoia* is broadly distributed across the Indo-Pacific islands, but is sister to New World boas [58]; such a biogeographic distribution is difficult to explain without considering the likelihood of oceanic dispersal. Another example is the presence of *Bolyeria* and *Casarea* on Round Island, Mauritius, while their sister *Xenophidion* is found in Southeast Asia [8]. These issues in tandem – the breakdown of the conceptual underpinnings of biogeographic methods and the high dispersal capabilities of snakes – suggest that the failure of Lagrange to reconstruct the snake biogeographic history indicates the fundamental inability of such methods to effectively broach the deep evolutionary histories of dispersive organisms.

In contrast to the Lagrange results, ‘naïve’ ASR methods (i.e., parsimony, ML, and Bayesian stochastic character mapping) reconstruct the ‘Serpentes’ ancestor as most likely originating on the Gondwanan Supercontinent (note, however, that reconstructions using the genetic topology disagree with those using the combined topologies, strongly reconstructing the ‘Serpentes’ ancestor as having originated on Laurasia – this is likely due in no small part to the lack of fossil information in the genetic analyses). This conclusion agrees with previous work suggesting a Gondwanan history for crown snakes, and in particular Scolecophidia [59], which is sister to all other crown snakes. Reconstructions for the ‘Total-Group’ node are more equivocal: the most parsimonious state for both the constrained and unconstrained topologies is a Laurasian origin, while ML and Bayesian methods reconstruct a Gondwanan origin as being only slightly more likely. Although the unambiguously Laurasian geographic distribution of a succession of anguimorphan outgroups supports a Laurasian origin for stem snakes [8], the ambiguity surrounding the ‘Total-Group’ node is likely also due to the Laurasian occurrence (specifically, North American) of the problematic early snake *Coniophis precedens*, which has been argued to represent the sister group to all other snakes [7]. Although this topological hypothesis is corroborated by our phylogenetic analyses, the validity of this argument hinges largely on whether all of the disarticulated elements referred to *Coniophis* are truly associated with a single species, a claim that requires further investigation. Future fossil discoveries and analyses may also potentially change our understanding of *Coniophis* and the deepest portions of the snake stem (e.g., the recent discovery of a jugal-bearing specimen of *Najash rionegrina* [6], one of the plesiomorphies previously thought to indicate the stemward position of *Coniophis precedens* [7]).

These results thus support a Gondwanan provenance for crown snakes, while also suggesting the possibility of a Laurasian origin for the snake total-group. Acceptance or rejection of this hypothesis necessarily relies on the future reevaluation of specimens referred to *C. precedens* and the discovery of additional early representatives of Pan-Serpentes.

### Divergence time estimation

**Figure 8:**
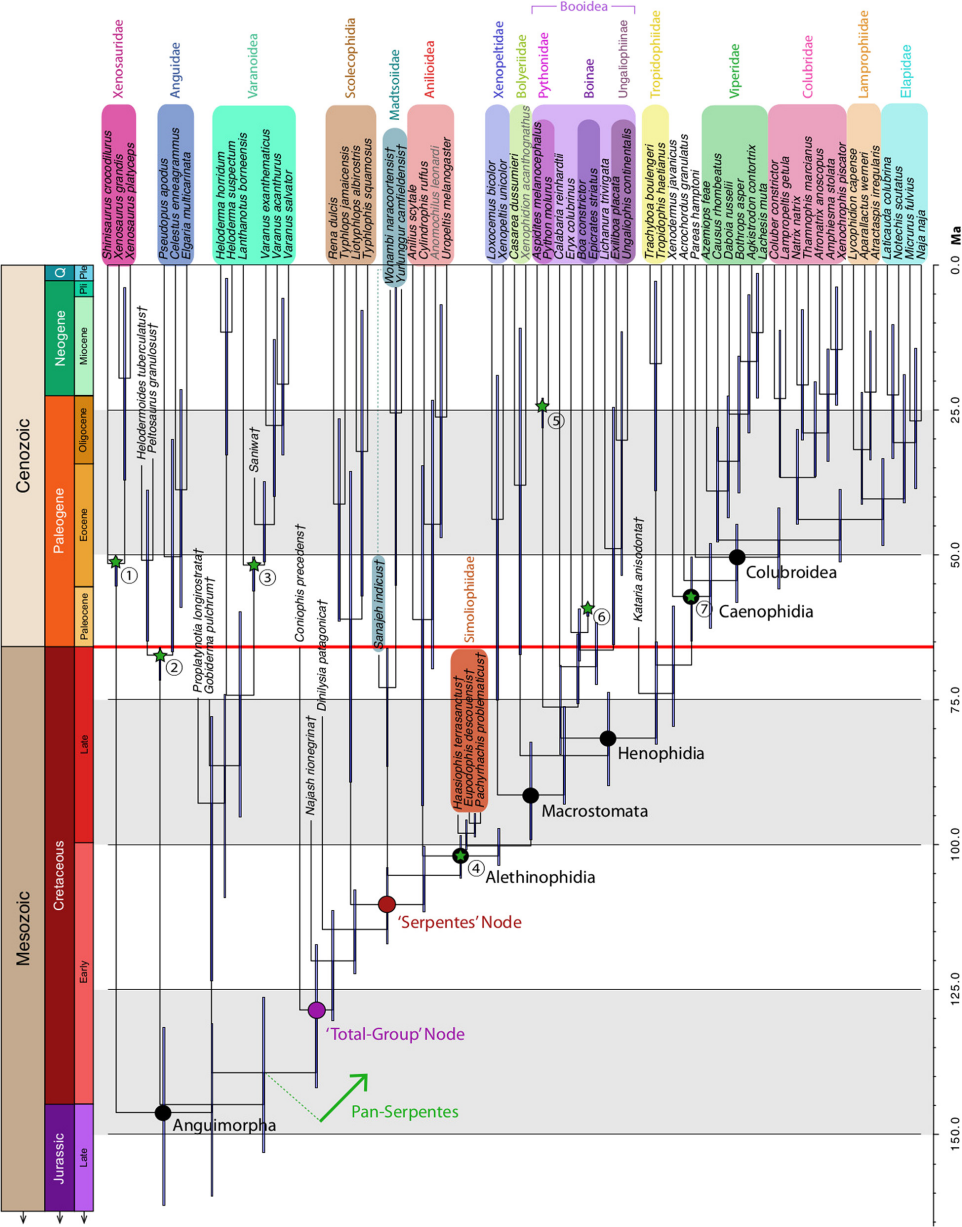
Divergence time tree inferred using the constrained topology. Divergence times inferred using the constrained tree in BEAST. Major crown clades are named, along with two extinct clades (Simoliophiidae and Madtsoiidae). The red line separating the Mesozoic and Cenozoic eras marks the Cretaceous-Paleogene (K-Pg) boundary at 66 Ma. Timescale is in millions of years. Circled numbers and green stars correspond to calibration dates outlined in Additional file 12. Colored boxes indicate major clades. Fossil taxa are marked with a dagger (†). Grayed taxa names indicate extant species that are included on the basis of phenotypic data only.

Divergence time trees were estimated for the genetic (see Additional file 6), unconstrained (see Additional file 7), and constrained trees (Figure 8). The constrained topology is presented because, unlike the other topologies, it preserves scolecophidian, anilioid, xenopeltid, booid, and tropidophiid + caenophidian monophyly. The following discussion, however, applies equally to all of the time-calibrated trees, with the exception of specific numbers regarding dates and their 95% highest posterior density intervals (HPDI), and when otherwise noted.

Pan-Serpentes is inferred to have originated ~128.5 Ma (mean age; HPDI [142.0, 117.2]), while crown snakes are inferred to have diverged ~110.3 Ma (HPDI [117.1, 104.0]). Given the error margins in this analysis, these events appear to have occurred in relatively quick succession during the late Early Cretaceous (specifically, during the Albian stage). The successive divergences of madtsoiids, pan-anilioids, simoliophiids, and pan-macrostomatans occurred in a remarkably rapid series of events between 105–95 Ma, with basal splits in crown macrostomatans following shortly thereafter (91.4 Ma; HPDI [99.2, 82.3]). The timing of these rapid basal divergences falls within the range of dates associated with the Cretaceous Terrestrial Revolution (125–80 Ma) [60], an interval when many familiar floral and faunal groups – such as mammals [61,62], birds [61,63], and angiosperms [64] – appear to have experienced accelerated and widespread diversification in terrestrial ecosystems. Our analyses suggest that snakes also experienced a burst of radiation in the mid-Cretaceous, and may have been participants in this significant macroevolutionary event.

The initial splits within Macrostomata appear to have occurred in the early Late Cretaceous, with the crown divergence between Pan-Booidea and the tropidophiid + caenophidian total-group following later (mean: 81.6 Ma; HPDI [89.8, 73.7]). The modern radiation of crown caenophidian snakes, however, seems to spring forth later in the Cenozoic, starting around 65–50 Ma, soon after the K-Pg Mass Extinction. Although it should be noted that the HPDI of the deepest divergence in Booidea crosses the K-Pg boundary, the widespread distribution and astonishing diversification of henophidian snakes – which was driven primarily by the radiation of the Colubroidea [65] – clearly occurred after the end-Cretaceous mass extinction in the combined divergence time trees. This result is in contrast to previous studies (e.g., Burbrink and Pyron [66]), which inferred a Paleogene origin for Colubroidea, but with confidence intervals crossing the K-Pg boundary. However, it should be noted that the clade definitions of Burbink and Pyron [66] differ slightly from ours; their crown ‘Colubroidea’ is equivalent to our crown ‘Caenophidia’. The age of our crown ‘Colubroidea’ (that is, *Pareas ,* viperids, colubrids, lamprophiids, and elapids) appears approximately as old as the corresponding clade in Burbrink and Pyron [66]. Our genetic divergence time tree infers a similar result to previous studies using only genomic data, with a post K-Pg origination date for Colubroidea, but with error margins crossing the boundary (mean: 64.1 Ma; HDPI [70.4, 57.8]). The inclusion of fossils as tip taxa thus clearly affects inferred divergence dates, and suggests that the early divergence dates for the most species-rich modern clade of snakes are younger than previously assumed. This radiation was likely driven by the sudden availability of niches left vacant by the catastrophic K-Pg extinction, mirroring the astounding radiation of placental mammals [67], crown birds [68], and several other surviving groups of squamates [69] in the early Cenozoic.

## Conclusions

**Figure 9:**
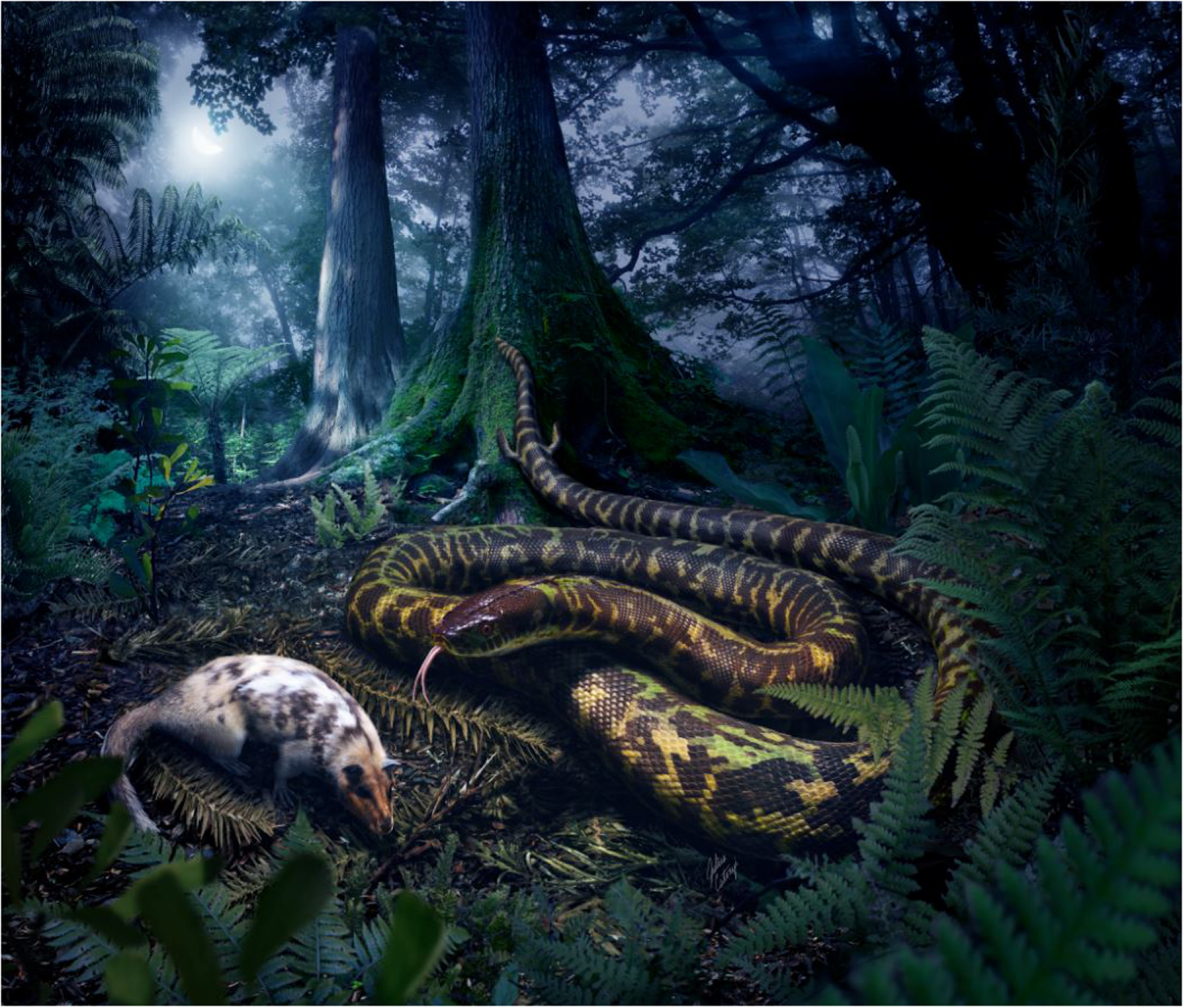
Reconstruction of the ancestral crown-group snake, based on this study. Artwork by Julius Csotonyi.

Based on our analyses, the ancestors of crown and total-group snakes were nocturnal stealth hunters that foraged widely for soft-bodied prey in warm, mild, well-watered, and well-vegetated ecosystems (Figure 9). Prey size was relatively small compared to prey regularly consumed by snakes exhibiting the macrostomatan condition, but large relative to the size of prey targeted ancestrally by non-snake lizards. It was unlikely that they employed constriction to subdue prey. The earliest snakes were likely active primarily on the ground surface (even if beneath cover), although they may have also exhibited semi-fossorial habits. Ancestral snakes are unequivocally inferred to have originated on land, rather than in aquatic settings. The biogeographic origin of snakes is less clear than their early ecology and behavior; however, our results suggest that the ancestor of crown snakes most likely originated on the Mesozoic supercontinent of Gondwana, and indicate the possibility that the ancestor of total-group snakes arose instead on Laurasia. A conclusive resolution of the biogeographic origin of total-group snakes will require both reevaluation of the controversial fossil snake *Coniophis precedens*, and the discovery of new fossils of stem-group snakes.

The snake total-group, or at least the *Coniophis*-node, is inferred to have arisen in the middle Early Cretaceous, with the crown originating about 20 million years later, during the Albian stage. A series of rapid divergences in their early evolutionary history suggests that snakes may have been participants in the hypothesized Early Cretaceous Terrestrial Revolution. Our results further suggest that henophidian diversity, which includes the bulk of extant snake species, radiated entirely after the K-Pg mass extinction.

These results paint the clearest picture yet of the early evolution of snakes, shedding light on their ecological, behavioral, biogeographic, and macroevolutionary origins. Both the ancestors of total-group and crown-group snakes were apparently similar in ecology and behavior to many basal macrostomatans surviving today. This conclusion, dependent on the inclusion of fossil stem snakes in our analysis, would be unexpected if only extant snakes were considered, given the sister-position of highly derived scolecophidians to all other extant crown snakes. Thus, the importance of fossil intermediates for illuminating macroevolutionary processes cannot be understated. Furthermore, our results demonstrate that the inclusion of phenotypic and fossil data can affect the inference of phylogenetic topologies, even when such data are vastly outnumbered by genetic sequence data. Fossils afford unprecedented glimpses into the grand tapestry of evolutionary history, and can inform inferences well beyond those that can be drawn from the fortuitous survivors comprising Earth’s modern biota. Transitional fossils are therefore invaluable for both phylogenetic analyses and for the accurate reconstruction of ancestral states.

## Methods

### Materials and data

#### Ancestral state reconstruction characters

Eleven novel characters describing ecology and habitat, feeding behavior, diel activity pattern, and tectonic plate occupation (see Additional file 1) were coded for 73 species – of which 15 are extinct – spanning the snake and other anguimorphan (outgroup) tree topology. Although our sample represents only a small percentage of the total extant diversity of snakes, the need to include fossils and phenotypic data precluded the inclusion of thousands of snake species in this study from a practical perspective. Future analyses would, of course, ideally sample a greater proportion of living snakes; however, even if we had examined every species of living snake, that sample might represent no more than a small fraction of the total diversity of a clade of such antiquity. Nevertheless, our dataset represents the most comprehensive sample combining genetic and phenotypic data to date.

Character codings were based on literature searches (see Additional file 8 for sources). All character codings are available in Nexus format on the Dryad Digital Depository. Although including both the ‘Tectonic Plate I’ and ‘Tectonic Plate II’ characters may be interpreted as pseudoreplication, the inclusion of both characters is intended to facilitate testing of the potential effects of using a simple binary character (‘Tectonic Plate I’) vs. a highly atomized, multi-state character (‘Tectonic Plate II’) during ancestral state reconstruction. The ‘Foraging Mode’ character refers to hunting strategy – that is, whether the species in question actively travels and forages for prey, largely remains sedentary and waits to ambush prey, or exhibits some combination of the two strategies. The ‘Prey Pursuit Method’ character then captures whether, upon detection of a prey item, the species in question employs an overt, charging attack, or a covert, stealthy approach and rapid strike (analogous to the contrast between the mode of hunting employed by cheetahs vs. leopards). The ‘Prey Preference’ character, in addition to being based on published natural history observations, is tied to tooth form; that is, teeth that are suited for crushing/piercing are assumed to belong to taxa that feed on prey items with relatively hard exoskeletons/exteriors, such as beetles, whereas teeth that are suited to prehension were assumed to belong to taxa that feed on relatively soft-bodied prey items, such as rodents and birds. This approach allowed us to code this character for fossil taxa in which tooth morphology, but not direct dietary information, is preserved. The ‘Prey Size’ character serves as a proxy for understanding the evolution of the unique macrostomatan condition – that is, a kinetic system enabling them to ingest intact prey items that are much larger than the head – within snakes (a capacity that evolved twice if tropidophiids are basal alethinophidians as genomic data suggest). As such, this character refers strictly to whether the species in question possesses the ability to swallow whole prey items that are larger than its head, and not to whether it also happens to consume prey that are smaller or subequal to head width (e.g., although *Boa constrictor* has been observed to opportunistically consume small prey such as mice, it would be scored as being able to swallow prey larger than its head). The remaining characters – ‘Diel Activity Pattern,’ ‘Biome’, ‘Prey Subdued by Constriction’, ‘Habitat Stratum’, and ‘Aquatic Habits’ – are self-explanatory. ‘Biome’ can admittedly be ambiguous, however, as it combines biotic (often botanical) associations with parameters of the physical environment, such as rainfall and temperature patterns. Reconstructions of ancestral ecologies would likely be more accurately served by scoring organisms for ‘Climate Zones’ rather than ‘Biomes’, so as to avoid confusing the former with the kinds of plants that currently happen to inhabit them [70] (e.g., while there were almost certainly semiarid climates in the Early Cretaceous, it is doubtful that they supported grasslands as they do today). As such, we would urge biologists who wish to consider these issues in the future to avoid this potential pitfall by considering past climate zones in lieu contemporary biome descriptions, especially for deep time reconstructions of ecological ancestral states.

Behavioral characters can be highly variable, and as such we applied a modal, and we believe repeatable, character-coding criterion. For example, diel activity patterns are widely understood to vary seasonally in snakes dwelling at higher latitudes; many normally nocturnal snakes in the arid American Southwest can be active during daylight hours, as weather permits, when emerging from hibernation in order to bask, mate, and feed. Nevertheless, those taxa are still regarded here as being ‘nocturnal’ so long as they exhibit that preference during most of their active seasons. The same subjective rationale was applied for modal state assignments to other behaviors, such as foraging and prey pursuit, and prey constriction. The identity of *Lichanura trivirgata* as a constrictor, for example, is undisputed by herpetologists despite considerable behavioral plasticity (e.g., individuals of the species have been observed to consume already dead mice without first constricting them), and is coded in our matrix as such. Commitment to aquatic habits and a particular stratum are arguably even more problematic due to their continuous variation, sometimes changing during the course of a snake’s lifetime. For instance, although individuals of *Lampropeltis getula* can occasionally be found climbing in low bushes, the species is generally described in the herpetological literature as “terrestrial.” In cases where our selected taxa were regularly described in the literature as exhibiting multimodal habits, they were accordingly scored as polymorphic. For instance, with regard to ‘Diel Activity Pattern,’ the viper *Causus rhombeatus* can apparently be found active at any time of day, and was accordingly scored as diurnal, crepuscular, and nocturnal to reduce potential bias in our ancestral state reconstructions.

Additionally, due to sampling limitations, certain clades in our dataset are represented by species that do not necessarily reflect the full diversity and disparity of their respective clades. The most notable example of this is our sampling of Scolecophidia: although scolecophidian snakes are found all over the world [71], our dataset includes only species found in North America and the West Indies, as a considerable amount of data has been gathered from these species due to their relative ease of access to researchers in the United States. This sampling bias may have an effect on the biogeographic reconstructions at our nodes of interest. Accordingly, all taxa were scored to represent the entire range of the genera to which they have been assigned in traditional taxonomies. For example, *Rena* (formerly *Leptoyphlops*) *dulcis* is found primarily in the southwestern United States and northern Mexico, but because *Leptotyphlops* as a clade can also be found throughout Central and South America, *R. dulcis* is coded in our matrix as both 0 and 1 (Laurasia and Gondwana).

#### Phenotypic and genetic data

The complete phylogenetic dataset included 766 phenotypic characters from the latest revision of the squamate dataset from the Assembling the Tree of Life (AToL) project [7,8] and 18,320 bp from 21 nuclear loci and one mitochondrial locus downloaded from the NBCI GenBank database (see Additional file 9). Nexus files for all datasets (phenotypic + ancestral state characters; genetic; combined) and a complete list of characters and character states are available on the Dryad Digital Repository.

Total genetic data coverage was 81.3% (excluding fossil taxa). In cases where genetic data were not available for the species sampled in the phenotypic dataset, genetic data were substituted from another species attributed to the same genus in traditional taxonomies (i.e., *Naja naja* and *Naja kaouthia*; *Rena dulcis* and *Rena humilis*; *Causus rhombeatus* and *Causus defilippi*). Genetic sequence data for our set of genes were unavailable for the two extant species *Anomochilus leonardi* and *Xenophidion acanthognathus.* Genetic data were aligned in *Clustal Omega* (v.1.1.0) [72] using default settings, and then inspected by eye in *BioEdit* (v.7.1.3.0) [73]. Model testing for each locus was conducted in PAUP* (v.4.0b10) [74] using the package *MrModelTest* (v.2.3) [75]. Under the Akaike Information Criterion, the best substitution model was determined to be GTR + I + G for all loci except *ZEB2*, for which the best model was HKY + I + G.

### Phylogenetic analyses

Phylogenetic trees were inferred using the following datasets: 1) the phenotypic data alone; 2) the genetic data alone; 3) the combined phenotypic and genetic data, unconstrained; and 4) the combined phenotypic and genetic data, constrained such that the interrelationships of certain major clades corresponded to those exhibited in the phenotypic-data-only tree topology (for a discussion and list of constraints implemented, see Additional file 10). The phenotype-only dataset was analyzed using both maximum parsimony and Bayesian methods, while the other three analyses were conducted using only Bayesian methods. In all cases, Xenosauridae (= *Shinisaurus crocodilurus*, *Xenosaurus grandis*, and *Xenosaurus platyceps*) was set as the monophyletic outgroup [8].

The dataset containing only phenotypic data was analyzed in PAUP* (v.4.0b10) [74] using a heuristic search algorithm with starting trees built using random stepwise addition with tree bisection and reconnection (TBR) branch swapping and twenty random addition sequence replicates. A strict consensus tree of the six most parsimonious trees (2,170 steps) was built (see Additional file 11). A nonparametric bootstrap search was conducted under the same heuristic search parameters for 1000 replicates, summarized as a 50% majority rule consensus tree (Figure 5). The same run parameters were used to build a tree using parsimony for the combined dataset: the most parsimonious tree consisted of 31,405 steps (see Additional file 12), and a 50% majority rule consensus tree was built from 1000 bootstrap replicates (Figure 6).

Bayesian phylogenetic analyses were run using *MrBayes* (v.3.2.2) [76] on the CIPRES Science Gateway [77]. The *Mkv* model [78] was used for the phenotypic data with gamma-distributed rate variation and variable coding. Sequence data were partitioned by gene, whereas phenotypic data were partitioned by number of character states (i.e., binary characters formed a partition, characters with three states formed a partition, etc.) to reflect implicit differences among rates of evolution for characters with more states vs. those with fewer. All analyses were run with a sampling frequency of 1000, two concurrent runs, and four Metropolis-coupled chains (*T* = 0.1). The phenotypic-data-only analysis was run for 20 million generations; all other datasets were run for 50 million generations. Model parameters (character state frequencies, substitution rates, gamma shape parameter, and proportion of invariable sites) were unlinked across all partitions, and rates were allowed to vary independently for all partitions. All analyses were checked for convergence using standard *MrBayes* diagnostics (e.g., PRSF < 0.01, mixing between chains > 20%) and *Tracer* (v.1.5) [79] (e.g., ESS > 200). A 25% relative burn-in was implemented for all summary statistics.

### Divergence time estimation

Divergence time trees were inferred using the genetic tree, the unconstrained tree, and the constrained tree. A maximum of seven nodal calibration points were used (six for the genetic tree; see Additional file 13 for list of calibration points and age-indicative fossils), along with tip calibration dates (see Additional file 14) for the unconstrained and constrained analyses. All calibrations follow the best practices protocols outlined by Parham *et al.* [80]. Beginning and ending dates for each geological period are defined by the standards of the International Commission on Stratigraphy (ICS). The three trees were first scaled such that the calibrated nodes matched their respective hard minimum ages using the *BLADJ* module in the *Phylocom* software package (v.4.2) [81]. The root age, which is required for *BLADJ*, was set at 150 Ma; this is the age of the earliest known crown squamates, including *Paramacellodus*, a stem-member of the scincomorph sister clade to all anguimorphs (including snakes) considered in this analysis [8].

The time-calibrated analyses were run in *BEAST* (v.1.8.0) [82] for 100 million generations on CIPRES. Calibration priors for both the nodal and tip calibration dates were set such that the youngest age of each geological period was set as the hard minimum age constraint offset for exponential distribution age priors; the scale parameter was then chosen such that 95% of the distribution volume was contained within the oldest age of the geological period. Tree operators (subtreeSlide, narrowExchange, wideExchange, and wilsonBalding) were switched off so that the analyses would optimize only node ages and not tree topology. For the genetic tree, the data were partitioned according to the 22 genes with unlinked substitution models, a single linked molecular clock model, and a linked tree model. For the unconstrained and constrained trees, the combined phenotypic and genetic data were divided into 27 partitions (22 gene partitions + 5 phenotypic partitions) with unlinked substitution models, a single molecular clock model, a single phenotypic clock model, and a linked tree model. The substitution models were set for the gene loci as specified in the original phylogenetic analyses, and the Lewis *Mk* model was used for the morphological partitions. Uncorrelated lognormal relaxed clock models were used with estimated rates. The birth-death serially sampled [83] speciation tree prior was used in all cases. Clock mean priors were set as diffuse gamma distributions (shape = 0.001, scale = 1000). All other priors were left with their default settings. Time-calibrated trees were summarized using *TreeAnnotator* (v.1.8.0), included with the BEAST software package, with a 25% burn-in.

### Ancestral state reconstruction

Ancestral states were inferred in all cases for the most recent common ancestor (MRCA) approximating the snake total-group (‘Total-Group’ node) and for the MRCA of crown snakes (‘Serpentes’ node). A nodal approximation for the snake total-group – which is certainly older than the divergence of *Coniophis precedens* from other pan-snakes – is necessary, as nodes and stem branches are not equivalent [84], but ancestral states can only be reconstructed at nodes. Ancestral state reconstruction (ASR) analyses were conducted under parsimony, maximum likelihood (ML), and Bayesian frameworks. *Mesquite* (v.2.75) [85] was used for parsimony ASR. The *R* (v. 2.15.1) [86] package *phytools* (v.0.3-93) [87], specifically the function *rerootingMethod*, was used for ML ASR. This function implements the Yang *et al.* [35] re-rooting method to estimate marginal ancestral states under a likelihood framework. For Bayesian stochastic character mapping [36,37], the *phytools* function *make.simmap* was used. These methods were chosen for their ability to incorporate uncertainty (i.e., missing data) and to take polymorphic states, which are extensive in our dataset, into account. It should be noted that these methods take missing data and polymorphic states into account by imposing a prior on the distribution of states for a given tip taxon. As a result, these states are not immutable during ancestral state estimation (as monomorphic states are) and may be overwritten – e.g., if taxon A is polymorphic for states 0 and 1, but happens to be nested with a clade in which all other clade members are monomorphic for state 0, the ASR process may reconstruct taxon A as exhibiting only state 0. Although this is obviously not ideal behavior, we believe the ability to include polymorphic data is preferable to excising such data from the analysis, as would be required in ‘traditional’ ASR methods such as *ace* [88]. Similarly, if a taxon in the analysis is coded as missing data (the ‘?’ state), the SIMMAP method will infer the most likely tip state for that taxon based on the available data (e.g., although all taxa exhibit a concrete tip state in Figure 6, some of those tip states – including all fossil taxa – are *inferred* states, not *coded* states).

All ancestral state reconstructions were performed on the time-calibrated genetic, unconstrained, and constrained topologies. For Bayesian stochastic character mapping, characters with missing data were initialized with a flat prior (all character states equally likely), whereas polymorphic characters were defined to have each polymorphic state be equally probable, with all other states exhibiting zero probability (e.g., a taxon coded 0&1 for a three-state character would have its prior set as [0.5, 0.5, 0]). The chi-squared log likelihood ratio test, using a significance level of 0.05, was used to determine which of the three available hierarchical models (ER, SYM, and ARD) was most appropriate for each character for each topology. For the genetic tree, the ER model was chosen for every character except ‘Foraging Mode’, ‘Diel Activity Pattern’, ‘Prey Preference’, ‘Habitat Stratum’, and ‘Aquatic Habits’, for which SYM was chosen for ‘Foraging Mode’ and ARD was chosen for all other characters. For the unconstrained tree, the ER model was chosen for every character. Finally, for the constrained tree, the ER model was chosen for every character except ‘Diel Activity Pattern’ and ‘Tectonic Plate I’, for which the SYM model was chosen. Bayesian stochastic mapping analyses were run for 5000 simulation replicates, with all other options set as default.

### Biogeographic reconstruction

In addition to using the naïve ancestral state reconstruction methods described in the previous section, the biogeographic method Lagrange [89] was used to reconstruct the history of the ‘Plate II’ tectonic plate character. The program *RASP* (v.3.0) [90] was used to implement Lagrange under the Dispersal-Extinction-Cladogenesis (DEC) model. The genetic, unconstrained, and constrained trees were each used as starting trees. All possible combinations of geographic ranges were allowed, and possible ranges were added automatically. Dispersal constraint matrices were built for six time intervals, based on tectonic plate movement: 0–3 MYA, 3–14 MYA, 14–50 MYA, 50–66 MYA, 66–94 MYA, and 94–133.27 (root age) MYA. Relative dispersal probabilities were based on tectonic plate reconstructions from the PALEOMAP project [91] using the following rules: 1) Connected landmasses are assigned a dispersal probability of 0.9; 2) Landmasses separated by an ocean of comparable size to the Atlantic/Indian/Tethys oceans are assigned a dispersal probability of 0.1; 3) Landmasses separated by an ocean of comparable size to the Pacific Ocean are assigned a dispersal probability of 0.01; and 4) Landmasses and close islands (e.g., the Caribbean islands and North America) are assigned a dispersal probability of 0.5. In cases where multiple rules are applicable, dispersal probabilities are multiplied; for instance, dispersal from North America to Australia in the Quaternary requires transversal across the Atlantic Ocean to the Eurasian landmass (0.1), then a jump from the Eurasian landmass to the Indo-Pacific islands (0.5), then a jump from the Indo-Pacific oceans to Australia (0.5), resulting in a relative dispersal probability of 0.025. Although dispersal between North America and Australia could also theoretically proceed across the Pacific Ocean, according to the aforementioned rules this would have a relative probability of 0.01 – in all such cases, the higher relative dispersal probability was used.

## Availability of supporting data

The data sets supporting the results of this article are available in the Dryad repository: https://datadryad.org/resource/doi:10.5061/dryad.7ct1n [92].

## Additional files

Additional file 1:
**List of characters and state descriptions for ancestral state reconstruction.**
Additional file 2:
**Genetic tree ASR results (MPS = Most Parsimonious State(s); ML = Maximum Likelihood).**
Additional file 3:
**Unconstrained tree ASR results (MPS = Most Parsimonious State(s); ML = Maximum Likelihood).**
Additional file 4:
**Constrained tree ASR results (MPS = Most Parsimonious State(s); ML = Maximum Likelihood).**
Additional file 5:
**Lagrange results for genetic, unconstrained, and constrained topologies.**
Additional file 6:
**Fossil-calibrated divergence time tree generated from the genetic dataset using the genetic tree.** The K-Pg boundary (66 MYA) is marked with a red line. Blue node bars denote 95% highest posterior density (HPD) intervals. Colored boxes indicate major clades. Colored lines indicate major clades from traditional taxonomies that do not resolve as monophyletic groups in this topology. Additional file 7:
**Fossil-calibrated divergence time generated from the combined phenotypic and genetic datasets using the unconstrained tree.** The K-Pg boundary (66 MYA) is marked with a red line. Blue node bars denote 95% highest posterior density (HPD) intervals. Colored boxes indicate major clades. Colored lines indicate major clades from traditional taxonomies that do not resolve as monophyletic groups in this topology. Additional file 8:
**List of natural history references used for coding ancestral state reconstruction characters.**
Additional file 9:
**GenBank accession numbers for genetic sequences used in this study.**
Additional file 10:
**Topology Constraints for Constrained Tree.**
Additional file 11:
**Strict consensus cladogram of the six most parsimonious trees from a heuristic search under a parsimony framework using the complete phenotypic dataset.** Colored boxes indicate major clades. Colored lines indicate major clades from traditional taxonomies that do not resolve as monophyletic groups in this topology. Additional file 12:
**The most parsimonious tree inferred from a heuristic search under a parsimony framework using the combined phenotypic + genetic dataset.** Colored boxes indicate major clades. Colored lines indicate major clades from traditional taxonomies that do not resolve as monophyletic groups in this topology. Additional file 13:
**Nodal calibration dates for divergence time analyses in** ***BEAST*** **.** Node marked with a * was not used for the genetic topology. Additional file 14:
**Fossil tip calibration dates for divergence time dating in** ***BEAST.***
