# Supplementary material for: The origin of snakes: revealing the ecology, behavior, and evolutionary history of early snakes using genomics, phenomics, and the fossil record
Source: BMC Evol Biol. 2015 May 20;15:87. doi: 10.1186/s12862-015-0358-5 (PMC4438441; doi:10.1186/s12862-015-0358-5)
Supplement: Additional file 1: — List of characters and state descriptions for ancestral state reconstruction. [file 12862_2015_358_MOESM1_ESM.pdf]

Additional File 1. List of characters and state descriptions for ancestral state reconstruction.

#	Character	States	
767	Diel Activity Pattern	0	Diurnal
		1	Crepuscular
		2	Nocturnal
768	Tectonic Plate I	0	Laurasia
		1	Gondwana
769	Tectonic Plate II	0	North American
		1	South American
		2	African
		3	Europe
		4	Asia
		5	India
		6	Australia
		7	Caribbean
		8	Indo-Pacific
770	Biome	0	Tropical-Subtropical Moist Broadleaf Forests
		1	Tropical-Subtropical Dry Broadleaf Forests
		2	Tropical-Subtropical Grasslands, Savannas, Shrublands
		3	Tropical-Subtropical Coniferous Forests
		4	Temperate Broadleaf Mixed Forests
		5	Temperate Grasslands, Savannas, Shrublands
		6	Mediterranean Forests, Woodlands, Shrublands
		7	Desert Xeric Shrublands
		8	Temperate Coniferous Forests
771	Foraging Mode	0	Ambush
		1	Widely Foraging
		2	Ambush and Widely Foraging
772	Prey Pursuit Method	0	Charge
		1	Stealth
773	Prey Subdued By Constriction	0	Absent
		1	Present
774	Prey Preference	0	Relatively Hard Exoskeletons, Teeth Suited to Crushing and Piercing
		1	Relatively Soft-Bodied Animals, Teeth Suited to Prehension
		2	Primarily Termites, Ants, Including Their Larvae and Eggs
775	Prey Size	0	Head Wider Than Prey
		1	Head Width Subequal to Prey Width
		2	Prey Much Wider Than Head
776	Habitat Strata	0	Fossorial
		1	Semi-Fossorial
		2	Terrestrial
		3	Semi-Arboreal or Saxicolus
		4	Arboreal
777	Aquatic Habits	0	Non-Aquatic (Terrestrial)
		1	Semi-Aquatic Freshwater
		2	Aquatic Freshwater
		3	Aquatic Brackish Water
		4	Aquatic Marine
