# Supplementary material for: The origin of snakes: revealing the ecology, behavior, and evolutionary history of early snakes using genomics, phenomics, and the fossil record
Source: BMC Evol Biol. 2015 May 20;15:87. doi: 10.1186/s12862-015-0358-5 (PMC4438441; doi:10.1186/s12862-015-0358-5)
Supplement: Additional file 2: — Genetic tree ASR results (MPS = Most Parsimonious State(s); ML = Maximum Likelihood). [file 12862_2015_358_MOESM2_ESM.pdf]

Additional file 2. Genetic tree ASR results (MPS = Most Parsimonious State(s); ML = Maximum Likelihood).

Character	State	Serpentes			Total Group		
		MPS	ML	SIMMAP	MPS	ML	SIMMAP
Diel Activity Pattern	0		0.3333	0.3384	-	-	-
	1		0.3333	6.00×10^{-4}	-	-	-
	2	X	0.3333	**0.6610**	-	-	-
Tectonic Plate I	0	X	**0.9670**	**0.9696**	-	-	-
	1		0.0330	0.0304	-	-	-
Tectonic Plate II	0	X	0.1111	0.1068	-	-	-
	1		0.1111	0.1112	-	-	-
	2		0.1111	0.1038	-	-	-
	3		0.1111	0.1146	-	-	-
	4		0.1111	0.1106	-	-	-
	5		0.1111	0.1110	-	-	-
	6		0.1111	0.1102	-	-	-
	7		0.1111	0.1160	-	-	-
	8		0.1111	0.1158	-	-	-
Biome	0	X	0.1111	0.1124	-	-	-
	1		0.1111	0.1098	-	-	-
	2		0.1111	0.1130	-	-	-
	3		0.1111	0.1172	-	-	-
	4		0.1111	0.1092	-	-	-
	5		0.1111	0.1098	-	-	-
	6		0.1111	0.1028	-	-	-
	7		0.1111	0.1146	-	-	-
	8		0.1111	0.1112	-	-	-
Foraging Mode	0		1.77×10^{-5}	0	-	-	-
	1	X	**0.9999**	**1**	-	-	-
	2		5.50×10^{-6}	0	-	-	-
Prey Pursuit Method	0		0.0039	0.0036	-	-	-
	1	X	**0.9961**	**0.9964**	-	-	-
Prey Subdued By Constriction	0	X	**0.9410**	**0.9376**	-	-	-
	1		0.0590	0.0624	-	-	-
Prey Preference	0		0.0008	0.0010	-	-	-
	1	X	**0.9744**	**0.9726**	-	-	-
	2	X	0.0247	0.0264	-	-	-
Prey Size	0		0.0039	0.0032	-	-	-
	1	X	**0.9814**	**0.9850**	-	-	-
	2		0.0147	0.0118	-	-	-
Habitat Strata	0	X	0.25	**0.8074**	-	-	-
	1		0.25	0.0838	-	-	-
	2		0.25	0.1088	-	-	-
	3		0.25	0	-	-	-
Aquatic Habits	0	X	0.20	**0.8296**	-	-	-
	1		0.20	0.1280	-	-	-
	2		0.20	0	-	-	-
	3		0.20	0.0182	-	-	-
	4		0.20	0.0242	-	-	-
