# Supplementary material for: The origin of snakes: revealing the ecology, behavior, and evolutionary history of early snakes using genomics, phenomics, and the fossil record
Source: BMC Evol Biol. 2015 May 20;15:87. doi: 10.1186/s12862-015-0358-5 (PMC4438441; doi:10.1186/s12862-015-0358-5)
Supplement: Additional file 3: — Unconstrained tree ASR results (MPS = Most Parsimonious State(s); ML = Maximum Likelihood). [file 12862_2015_358_MOESM3_ESM.pdf]

Additional file 3. Unconstrained tree ASR results (MPS = Most Parsimonious State(s); ML = Maximum Likelihood).

Character	State	Serpentes			Total Group		
		MPS	ML	SIMMAP	MPS	ML	SIMMAP
Diel Activity Pattern	0		0.0007	0.0482		0.0076	0.1544
	1		0.0001	0.0260		0.0006	0.1238
	2	X	**0.9992**	**0.9258**	X	**0.9919**	**0.7218**
Tectonic Plate I	0		0.1399	0.1368	X	0.4728	0.4618
	1	X	**0.8601**	**0.8632**		**0.5272**	**0.5382**
Tectonic Plate II	0		0.0603	0.0572	X	0.3135	0.3102
	1	X	**0.7489**	**0.7608**		**0.4852**	**0.4926**
	2		0.0028	0.0018		0.0106	0.0100
	3		0.0454	0.0410		0.1166	0.1134
	4		0.0153	0.0162		0.0126	0.0114
	5		0.0039	0.0026		0.0107	0.0108
	6		0.0036	0.0032		0.0107	0.0112
	7		0.1172	0.1146		0.0295	0.0304
	8		0.0026	0.0026		0.0105	0.0100
Biome	0	X	0.1111	**0.9990**	X	0.1111	**0.9902**
	1		0.1111	0		0.1111	0.0008
	2		0.1111	0.0002		0.1111	0.0034
	3		0.1111	0.0002		0.1111	0.0016
	4		0.1111	0		0.1111	0.0006
	5		0.1111	0.0002		0.1111	0.0014
	6		0.1111	0.0002		0.1111	0.0004
	7		0.1111	0.0002		0.1111	0.0008
	8		0.1111	0		0.1111	0.0008
Foraging Mode	0		0.0001	0.0002		0.0011	0.0018
	1	X	**0.9998**	**0.9996**	X	**0.9985**	**0.9974**
	2		8.42×10^{-5}	0.0002		0.0004	0.0008
Prey Pursuit Method	0		0.0027	0.0042		0.0229	0.0262
	1	X	**0.9973**	**0.9958**	X	**0.9771**	**0.9738**
Prey Subdued By Constriction	0	X	**0.8087**	**0.8002**	X	**0.9226**	**0.9198**
	1		0.1913	0.1998		0.0774	0.0802
Prey Preference	0		3.91×10^{-6}	0		7.04×10^{-6}	0
	1	X	**0.9999**	**1**	X	**0.9999**	**1**
	2		0.0001	0		1.15×10^{-6}	0
Prey Size	0		0.0002	0		0.0090	0.0086
	1	X	**0.9992**	**0.9996**	X	**0.9904**	**0.9912**
	2		0.0006	0.0004		0.0005	0.0002
Habitat Strata	0	X	0.0621	0.0632	X	0.0322	0.0310
	1		0.0059	0.0072		0.0061	0.0082
	2	X	**0.9291**	**0.9272**	X	**0.9552**	**0.9546**
	3		0.0029	0.0024		0.0064	0.0062
Aquatic Habits	0	X	**0.9989**	**0.9986**	X	**0.9994**	**0.9994**
	1		6.96×10^{-6}	0		6.91×10^{-5}	0.0002
	2		6.02×10^{-6}	0		4.93×10^{-5}	0
	3		6.02×10^{-6}	0		4.93×10^{-5}	0.0004
	4		0.0011	0.0014		0.0005	0
