# Supplementary material for: The origin of snakes: revealing the ecology, behavior, and evolutionary history of early snakes using genomics, phenomics, and the fossil record
Source: BMC Evol Biol. 2015 May 20;15:87. doi: 10.1186/s12862-015-0358-5 (PMC4438441; doi:10.1186/s12862-015-0358-5)
Supplement: Additional file 4: — Constrained tree ASR results (MPS = Most Parsimonious State(s); ML = Maximum Likelihood). [file 12862_2015_358_MOESM4_ESM.pdf]

Additional file 4. Constrained tree ASR results (MPS = Most Parsimonious State(s); ML = Maximum Likelihood).

Character	State	Serpentes			Total Group		
		MPS	ML	SIMMAP	MPS	ML	SIMMAP
Diel Activity Pattern	0		0	0.0106		0	0.0746
	1		0	0.0128		0	0.0910
	2	X	**1**	**0.9766**	X	**1**	**0.8344**
Tectonic Plate I	0		0.1212	0.1222	X	0.4436	0.4418
	1	X	**0.8788**	**0.8778**		**0.5564**	**0.5582**
Tectonic Plate II	0	X	0.0378	0.0820	X	0.1850	0.1932
	1	X	**0.5482**	**0.5768**		**0.4443**	**0.5118**
	2		0.0050	0.0092		0.0143	0.0198
	3	X	0.0050	0.0570		0.0144	0.1080
	4	X	0.3647	0.0396		0.2806	0.0336
	5	X	0.0206	0.0154		0.0177	0.0228
	6		0.0060	0.0126		0.0145	0.0202
	7		0.0073	0.1982		0.0148	0.0688
	8		0.0054	0.0092		0.0144	0.0218
Biome	0	X	0.1111	**0.9974**	X	0.1111	**0.9856**
	1		0.1111	0.0012		0.1111	0.0018
	2		0.1111	0.0002		0.1111	0.0034
	3		0.1111	0.0002		0.1111	0.0012
	4		0.1111	0		0.1111	0.0006
	5		0.1111	0.0006		0.1111	0.0046
	6		0.1111	0.0002		0.1111	0.0010
	7		0.1111	0		0.1111	0.0006
	8		0.1111	0.0002		0.1111	0.0012
Foraging Mode	0		0.0002	0		0.0028	0.0022
	1	X	**0.9998**	**1**	X	**0.9965**	**0.9966**
	2		6.65×10^{-5}	0		0.0008	0.0012
Prey Pursuit Method	0		0.0022	0.0026		0.0248	0.0266
	1	X	**0.9978**	**0.9974**	X	**0.9752**	**0.9734**
Prey Subdued By Constriction	0	X	**0.7124**	**0.7078**	X	**08724**	**0.8668**
	1		0.2876	0.2922		0.1276	0.1332
Prey Preference	0		9.51×10^{-6}	0.0002		0.0002	0.0002
	1	X	**0.9997**	**0.9990**	X	**0.9998**	**0.9998**
	2		0.0003	0.0008		4.25×10^{-6}	0
Prey Size	0		0.0011	0.0014		0.0561	0.0586
	1	X	**0.9983**	**0.9984**	X	**0.9430**	**0.9402**
	2		0.0006	0.0002		0.0009	0.0012
Habitat Strata	0	X	0.2563	0.2584	X	0.1378	0.1402
	1		0.0125	0.0104		0.0118	0.0112
	2	X	**0.7266**	**0.7254**	X	**0.8318**	**0.8294**
	3		0.0046	0.0058		0.0186	0.0192
Aquatic Habits	0	X	**0.9999**	**1**	X	**0.9997**	**0.9998**
	1		2.75×10^{-6}	0		8.27×10^{-5}	0
	2		2.45×10^{-6}	0		6.46×10^{-5}	0
	3		2.45×10^{-6}	0		6.46×10^{-5}	0
	4		4.79×10^{-5}	0		8.18×10^{-5}	0.0002
