# Supplementary material for: The origin of snakes: revealing the ecology, behavior, and evolutionary history of early snakes using genomics, phenomics, and the fossil record
Source: BMC Evol Biol. 2015 May 20;15:87. doi: 10.1186/s12862-015-0358-5 (PMC4438441; doi:10.1186/s12862-015-0358-5)
Supplement: Additional file 6: — Fossil-calibrated divergence time tree generated from the genetic dataset using the genetic tree. The K-Pg boundary (66 MYA) is marked with a red line. Blue node bars denote 95% highest posterior density (HPD) intervals. Colored boxes indicate major clades. Colored lines indicate major clades from traditional taxonomies that do not resolve as monophyletic groups in this topology. [file 12862_2015_358_MOESM6_ESM.pdf]

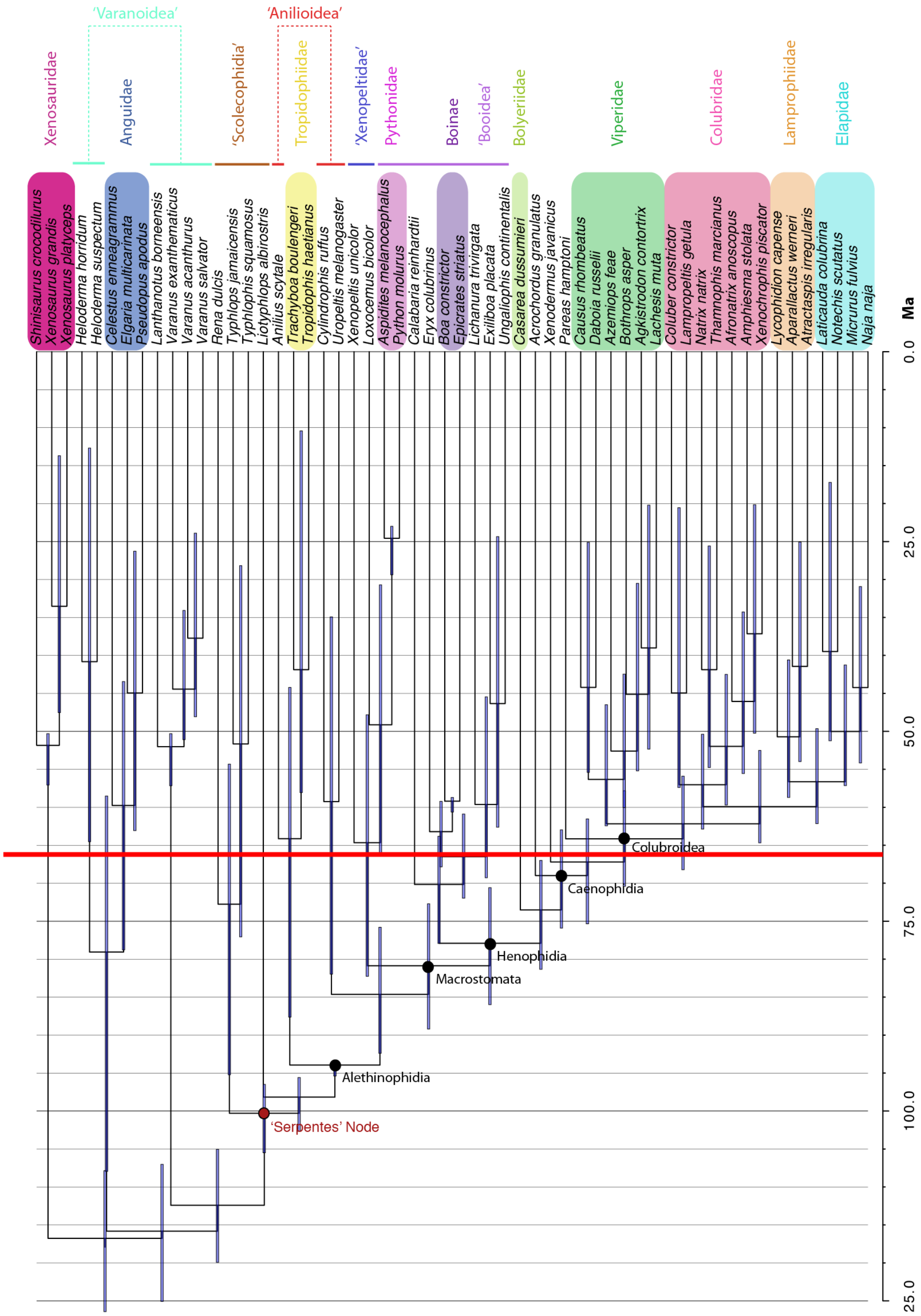

Additional file 6. Fossil-calibrated divergence time tree generated from the genetic dataset using the genetic tree. The K-Pg boundary (66 MYA) is marked with a red line. Blue node bars denote 95% highest posterior density (HPD) intervals. Colored boxes indicate major clades. Colored lines indicate major clades from traditional taxonomies that do not resolve as monophyletic groups in this topology.
