# Supplementary material for: The origin of snakes: revealing the ecology, behavior, and evolutionary history of early snakes using genomics, phenomics, and the fossil record
Source: BMC Evol Biol. 2015 May 20;15:87. doi: 10.1186/s12862-015-0358-5 (PMC4438441; doi:10.1186/s12862-015-0358-5)
Supplement: Additional file 10: — Topology Constraints for Constrained Tree. [file 12862_2015_358_MOESM10_ESM.pdf]

Additional file 10. Topology Constraints for Constrained Tree.

The constraints imposed for the constrained tree topology were chosen to enforce certain nodes inferred by the parsimony analysis of the phenotypic dataset. In some instances of discordance between morphology vs. molecules where the phenotypic support was weak – such as the position of the pareatid *Pareas hamptoni* among colubroid snakes – no constraints were defined, allowing the topology to be freely inferred from the combined genetic and phenotypic data. In other cases in which relationships were uncertain – for example, whether bolyeriids or booids are more closely related to caenophidians – the tree was left unconstrained. We preferred clades emerging from the phenotypic dataset when genetic data inferred alternative topologies marked by pronounced branch-length asymmetry. For example, we constrained Scolecophidia to be monophyletic even though sequence data strongly supported one of the two species of anomalepidid scolecophidians in the analysis (*Liotyphlops albirostris*) as being closer to alethinophidian snakes than is the other (*Typhlophis squamosus*). According to sequence data, Scolecophidia would be paraphyletic and Anomalepididae polyphyletic. Nevertheless, these internested clades are strongly supported by phenotypic data (100% and 99% bootstraps, respectively) and several characters, with 1.00 consistency indices. Anomalepidids, for example, are supported by at least two synapomorphies – relating to the role of the prefrontal in the upper jaw and the supraoccipital in the braincase – that are unique and unreversed among reptiles. Because the short branch connecting *Liotyphlops* to alethinophidians was only 1/25th of the long-branch connecting that node to extant *Liotyphlops*, we elected to constrain scolecophidian and anomalepidid monophyly.

In addition to the constraints mentioned above, the following constraints were implemented: **Xenosauridae** (*Xenosaurus* + *Shinisaurus*) as a monophyletic outgroup; and an ingroup composed of **Anguidae** = (*Elgaria* + *Celestus*) (*Helodermoides* + *Peltosaurus*) (*Pseudopus*); **Varanoidea =** (*Heloderma*) + (*Lanthanotus* + *Varanus*); **Scolecophidia; Cylindrophiidae** = *Anilius* ((*Cylindrophis* (*Anomochilus* + *Uropeltis*)); **Macrostomata** = (Xenopeltidae + Bolyeriidae + Booidea, + *Kataria* + Tropidophiidae + Caenophidia); **Xenopeltidae** = (*Xenopeltis* + *Loxocemus*); an unnamed clade including **Bolyeriidae** + **Henophidia; Bolyeriidae** = (*Casarea* + *Xenophidion*); **Henophidia** = (Booidea + *Kataria* + Tropidophiidae + Caenophidia); **Booidea** = Pythonidae (*Aspidites* + *Python*) + Boidae ((*Exiliboa* + *Ungaliophis*) + (*Boa* + *Epicrates*) + *Lichanura* + *Eryx* + *Calabaria*); a unnamed clade including **Tropidophiidae** (= *Tropidophis* + *Trachyboa*) + **Caenophidia** (**=** *Xenodermus* + *Acrochordus* + Colubroidea). Ambiguity regarding the placement of the extinct stem varanoids *Proplatynotia longirostrata* and *Gobiderma pulchrum* (which likely result from missing data) was resolved by constraining *P. longirostrata* and *G. pulchrum* to be successive sisters to crown Varanoidea (*Heloderma* + Varanidae) (i.e., *P. longirostrata*(*G. pulchrum*(Varanoidea))), as in the strict parsimony consensus tree.
