# Supplementary material for: The origin of snakes: revealing the ecology, behavior, and evolutionary history of early snakes using genomics, phenomics, and the fossil record
Source: BMC Evol Biol. 2015 May 20;15:87. doi: 10.1186/s12862-015-0358-5 (PMC4438441; doi:10.1186/s12862-015-0358-5)
Supplement: Additional file 11: — Strict consensus cladogram of the six most parsimonious trees from a heuristic search under a parsimony framework using the complete phenotypic dataset. Colored boxes indicate major clades. Colored lines indicate major clades from traditional taxonomies that do not resolve as monophyletic groups in this topology. [file 12862_2015_358_MOESM11_ESM.pdf]

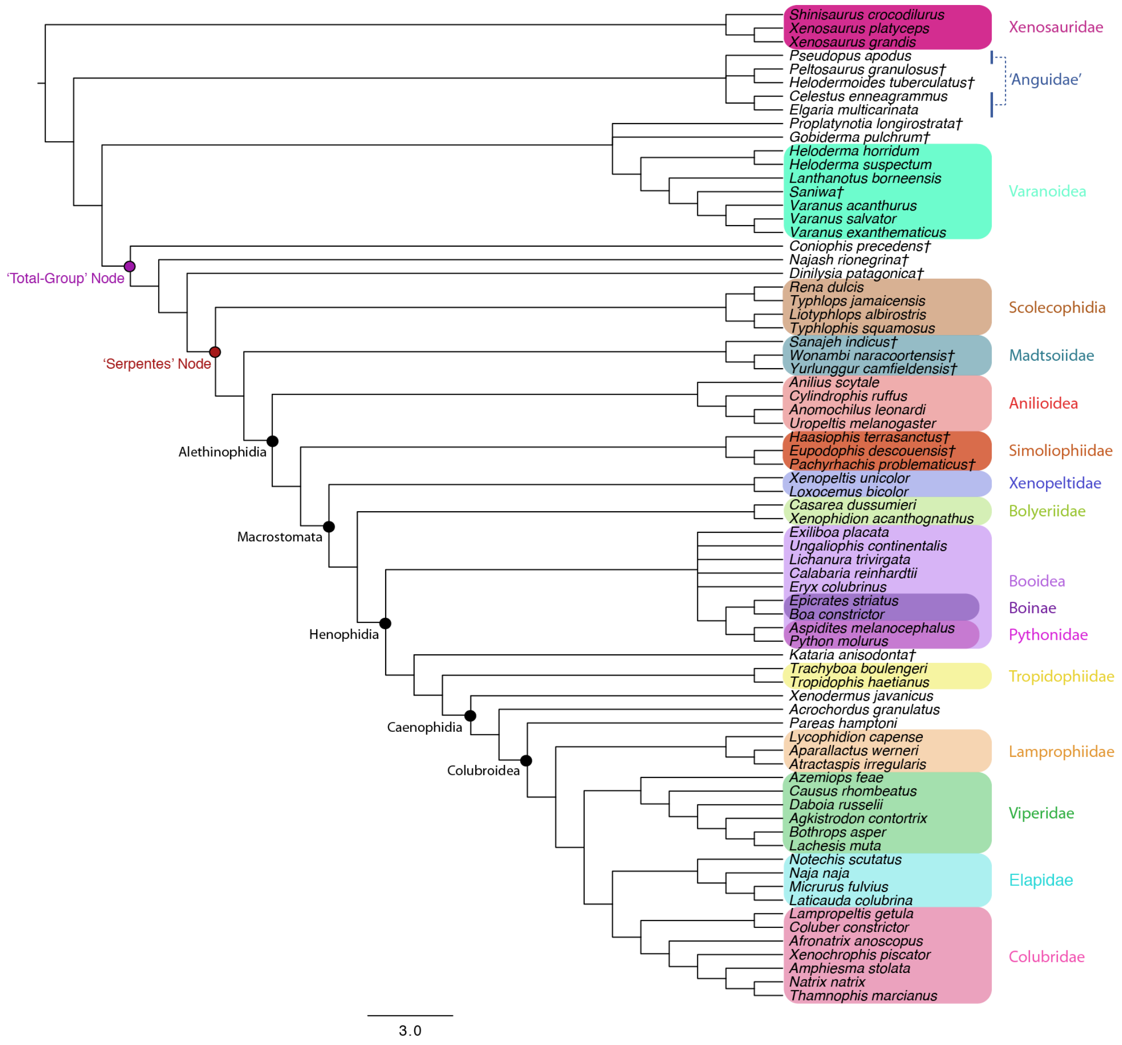

Additional file 11. Strict consensus cladogram of the six most parsimonious trees from a heuristic search under a parsimony framework using the complete phenotypic dataset. Colored boxes indicate major clades. Colored lines indicate major clades from traditional taxonomies that do not resolve as monophyletic groups in this topology.
