# Supplementary material for: The origin of snakes: revealing the ecology, behavior, and evolutionary history of early snakes using genomics, phenomics, and the fossil record
Source: BMC Evol Biol. 2015 May 20;15:87. doi: 10.1186/s12862-015-0358-5 (PMC4438441; doi:10.1186/s12862-015-0358-5)
Supplement: Additional file 13: — Nodal calibration dates for divergence time analyses in BEAST . Node marked with a * was not used for the genetic topology. [file 12862_2015_358_MOESM13_ESM.pdf]

Additional file 13. Nodal calibration dates for divergence time analyses in *BEAST*. Node marked with a * was not used for the genetic topology.

Node Number	Node Name	Age-Indicative Fossil	Geological Period	Age Range (in MYA)	Reference
1	*Shinisaurus* + *Xenosaurus*	*Entomophontes*	Early Eocene	55.4-50.3	Smith and Gauthier 2013
2*	Anguidae + Glyptosaurinae	*Odaxosaurus*	Late Cretaceous (Maastrichtian)	72.1-66.0	Estes 1964
3	*Lanthanotus* + *Varanus*	*Saniwa*	Early Eocene	55.4-50.3	Smith and Gauthier 2013
4	Alethinophidia	*Haasiophis*	Late Cretaceous (Cenomanian)	99.6-93.5	Tchernov *et al.* 2000
5	Pythonidae	*Morelia*	Late Oligocene	28.4-23.0	Scanlon 2001
6	Boinae	*Titanoboa*	Middle Paleocene	61.7-58.7	Head *et al.* 2009
7	Caenophidia	*Procerophis*	Early Eocene	55.4-50.3	Rage *et al.* 2008; Sanders *et al.* 2010

References

Estes R. Fossil Vertebrates from the Late Cretaceous Lance Formation, Eastern Wyoming. *Publications in Geological Sciences.* Vol. 49. Berkeley, California: Univ. of California Press; 1964.

Head JJ, Bloch JI, Hastings AK, Bourque JR, Cadena EA, Herrera FA, Polly PD, Jaramillo CA. Giant boid snake from the Palaeocene neotropics reveals hotter past equatorial temperatures. *Nature*. 2009;457:715-718.

Rage J-C, Folie A, Rana RS, Singh H, Rose KD, Smith T. A diverse snake fauna from the early Eocene of Vastan Lignite Mine, Gujarat, India. *Acta Palaeont Pol*. 2008;53:391-403.

Sanders KL, Mumpuni, Hamidy A, Head JJ, Gower, DJ. Phylogeny and divergence times of filesnakes (Acrochordus): inferences from morphology, fossils and three molecular loci. *Mol Phylo Evol* 2010;56:857-867.

Scanlon JD. *Montypythonoides*: the Miocene snake *Morelia riversleighensis* (Smith & Plane, 1985) and the geographical origin of pythons. *Mem Assoc Austr Palaeont*. 2001;25:1-35.

Smith KT, Gauthier JA. Early Eocene Lizards of the Wasatch Formation near Bitter Creek, Wyoming: Diversity and Paleoenvironment during an Interval of Global Warming. *Bull Peabody Mus Nat Hist*. 2013;54:135-230.

Tchernov E, Rieppel O, Zaher H, Polcyn MJ, Jacobs LL. 2000. A fossil snake with limbs. *Science* **287**:2010-2012
