# Supplementary material for: The origin of snakes: revealing the ecology, behavior, and evolutionary history of early snakes using genomics, phenomics, and the fossil record
Source: BMC Evol Biol. 2015 May 20;15:87. doi: 10.1186/s12862-015-0358-5 (PMC4438441; doi:10.1186/s12862-015-0358-5)
Supplement: Additional file 14: — Fossil tip calibration dates for divergence time dating in BEAST. [file 12862_2015_358_MOESM14_ESM.pdf]

**Additional file 14.** Fossil tip calibration dates for divergence time dating in *BEAST*.

| Fossil Taxon | Geological Period | Age Range (in MYA) | Distribution Mean | Reference |
|---|---|---|---|---|
| *Coniophis precedens* | Maastrichtian | 72.1 – 66.0 | 2.035 | Szyndlar and Rage 2003 |
| *Dinilysia patagonica* | Santonian-Campanian | 86.3 – 72.1 | 4.74 | Scanferla and Canale 2007; Albino 2007 |
| *Eupodophis descouensis* | Cenomanian | 100.5 – 93.9 | 2.2 | Rage and Escullié 2000; Rieppel and Head 2004 |
| *Gobiderma pulchrum* | Campanian | 83.6 – 72.1 | 3.84 | Eberth 2011 |
| *Haasiophis terrasanctus* | Cenomanian | 100.5 – 93.9 | 2.2 | Rieppel *et al.* 2003 |
| *Helodermoides tuberculatus* | Chadronian | 37.2 – 33.9 | 1.1 | Douglass 1903 |
| *Kataria anisodonta* | Danian | 66.0 – 61.6 | 1.47 | Scanferla *et al.* 2013 |
| *Najash rionegrina* | Cenomanian | 100.5 – 93.9 | 2.2 | Apesteguía and Zaher 2006 |
| *Pachyrhachis problematicus* | Cenomanian | 100.5 – 93.9 | 2.2 | Lee and Caldwell 1998 |
| *Peltosaurus granulosus* | Orellan | 33.9 – 33.3 | 0.2 | Gilmore 1928 |
| *Proplatynotia longirostrata* | Campanian | 83.6 – 72.1 | 3.84 | Eberth 2011 |
| *Sanajeh indicus* | Maastrichtian | 72.1 – 66.0 | 2.035 | Wilson *et al.* 2010 |
| *Saniwa* | Paleocene-Eocene | 63.3 – 33.9 | 9.815 | Leidy 1870 |
| *Wonambi naracoortensis* | Late Oligocene-Late Pleistocene | 28.1 – 0.0117 | 9.377 | Scanlon and Lee 2000 |
| *Yurlunggur* | Late Oligocene-Late Pleistocene | 28.1 – 0.0117 | 9.377 | Scanlon 2006 |

## References


Albino A. Lepidosauromorphs. In: Gasparini Z, Salgado L, Coria RA, editors. *Patagonian Mesozoic Reptiles*. Bloomington & Indianapolis, Indiana: Indiana University; 2007. p. 87-115.

Douglass E. New vertebrates from the Montana Tertiary. *Ann Carnegie Mus*. 1903;2:145-199.

Apesteguía S, Zaher H. Cretaceous terrestrial snake with robust hindlimbs and a sacrum. *Nature.* 2006;440:1037-1040.

Eberth DA. Depositional environments and fades transitions of dinosaur-bearing Upper Cretaceous redbeds at Bayan Mandahu (Inner Mongolia, People's Republic of China). *Can J Earth Sci*. 2011;30:2196-2213.

Gilmore CW. Fossil lizards of North America. *Mem Natl Acad Sci*. 1928;22:1-201.

Lee MSY, Caldwell MW. Anatomy and relationships of Pachyrhachis problematicus, a primitive snake with limbs. *Phil Trans Roy Soc London B*. 1998;353:1521-1552.

Leidy J. Descriptions of Emys jaenesi, E. haydeni, Baena arenosa, and Saniwa ensidens. *P Am Philos Soc*. 1870:123-124.

Rage J-C, Escuillié F. Un nouveau serpent bipède du Cénomanien (Crétacé). Implications phylétiques. *C R Acad Sci.* 2000;330:513-520.

Rieppel O, Head JJ. New specimens of the fossil snake genus Eupodophis Rage & Escuillié, from Cenomanian (Late Cretaceous) of Lebanon. *Mem Soc Ital Sci Nat Mus Civ Stor Nat Milano*. 2004;32:1-26.

Rieppel O, Zaher H, Tchernov E, Polcyn MJ. The anatomy and relationships of Haasiophis terrasanctus, a fossil snake with well developed hind limbs from the mid-Cretaceous of the Middle East. *J Paleont.* 2003;77:536-558.

Scanferla A, Canale JI. The youngest record of the Cretaceous snake genus Dinilysia (Squamata, Serpentes). *S Am J Herpetol*. 2007;2:76-81.

Scanferla A, Zaher H, Novas FE, de Muizon C, Céspedes R. A new snake skull from the Paleocene of Bolivia sheds light on the evolution of macrostomatans. *PLoS ONE.* 2013;8:e57583. doi: 10.1371/journal.pone.0057583.

Scanlon JD. Skull of the large non-macrostomatan snake Yurlunggur from the Australian Oligo-Miocene. *Nature.* 2006;439:839-842.

Scanlon JD, Lee MSY. The Pleistocene serpent Wonambi and the early evolution of snakes. *Nature.* 2000;403:416-420.

Szyndlar Z, Rage J-C. *Non-erycine Booidea from the Oligocene and Miocene of Europe.* Kraków, Poland: Institute of Systematics and Evolution of Animals, Polish Academy of Sciences; 2003.

Wilson JA, Mohabey D, Peters S, Head JJ. Predation upon hatchling sauropod dinosaurs by a new basal snake from the Late Cretaceous of India. *PLoS Biol*. 2010;8:e1000322. doi: 10.1371/journal.pbio.1000322.
